# Comparison on different traditional Chinese medicine therapies for chronic hepatitis B liver fibrosis

**DOI:** 10.3389/fphar.2022.943063

**Published:** 2022-08-10

**Authors:** Yun-kai Dai, Hai-na Fan, Yong-hong Hu, Zhi-min Zhao, Chenghai Liu

**Affiliations:** ^1^ Institute of Liver Diseases, Shuguang Hospital Affiliated to Shanghai University of Traditional Chinese Medicine, Shanghai, China; ^2^ Shanghai Key Laboratory of Traditional Chinese Clinical Medicine, Shanghai, China; ^3^ Key Laboratory of Liver and Kidney Diseases, Ministry of Education, Shanghai, China

**Keywords:** traditional Chinese medicines, chronic hepatitis B liver fibrosis, randomized controlled trials, clinical medication, surface under the cumulative ranking curve

## Abstract

**Background and Aims:** Although different kinds of traditional Chinese medicines could reportedly improve the efficacy of antiviral therapy on liver fibrosis caused by HBV, the problem of clinicians on how to choose the appropriate treatment strategies for the patients fails to be solved. This study aims at comparing and ranking different traditional Chinese medicine (TCM) therapies in the treatment of liver fibrosis due to chronic hepatitis B (CHB).

**Methods:** Eight electronic databases were searched from their establishment to 17 Aug 2021. All included data and pooled odds ratio were used for network meta-analysis (NMA) and statistical analysis. The consistency was evaluated by the node-splitting analysis. The stability of results and source of heterogeneity were tested by sensitivity analysis. Different treatment strategies (regimens) in this network meta-analysis were ranked with the aid of surface under the cumulative ranking curve (SUCRA) probability value.

**Results:** A total of 29 articles with 3,106 sufferers were recruited in this NMA. Results of SUCRA value rankings indicated that Fuzheng Huayu therapy or combined with entecavir had preferable effects in improving the clinical efficacy, recovering the level of hyaluronic acid, IV-C, ALT, ALB, and TBil, relieving the TCM symptoms including hypochondriac pain and poor appetite, regaining the width of portal vein and thickness of spleen, and lessening side effects. Apart from these, Ziyin Shugan therapy or combined with ETV could also be suitable to regain the level of laminin, PC-III, and AST, relieve fatigue and HBV-DNA conversion.

**Conclusion:** This NMA confirmed the efficacy and safety of different treatment therapies for improving CHB liver fibrosis, including the serum biomarkers of live fibrosis and serum parameters for liver function, TCM symptoms, imaging indexes, HBV-DNA conversion rate, which offered the TCM practitioners crucial reference value on clinical medication.

## Introduction

Chronic hepatitis B (CHB), characterized by persistent hepatitis B virus (HBV) infection, is a serious infectious disease with high morbidity and mortality (He et al., 2016; Luo et al., 2016). Liver fibrosis is characterized by excessive accumulation of the collagen extracellular matrix (ECM) (Suk and Kim, 2015). As is well known, CHB is one of the most important etiologies for liver fibrosis, which could develop into cirrhosis. It is reported that 3% of CHB sufferers have decompensated liver cirrhosis every year. This aggravation could shorten 5-years cumulative survival time for patients with CHB (Ren et al., 2020). Therefore, inhibiting or regressing liver fibrosis is as a crucial strategy for CHB as anti-HBV replication.

Currently, nucleos (t) ide analogs, such as entecavir (ETV), tenofovir dipivoxil, and adefovir dipivoxil, are mainly applied to clinical treatment, whose therapeutic mechanism is involved in the continuous suppression of the activity of HBV-DNA reverse transcriptase. Although these analogs, to some extent, can alleviate CHB fibrosis, they require the sufferers to take medicine for long time, and their efficacies of antifibrosis are limited. A research finding showed that the improvement rate of liver fibrosis was about 40% after 1 year of application of antiviral drugs (Schiff et al., 2008). Meanwhile, HBV patients with serious fibrosis received antiviral treatment for 5–10 years, and only about one-third of the patients failed to improve their liver fibrosis (Chang et al., 2010).

Due to the complexity of pathological mechanism of liver fibrosis, there are currently no FDA-approved biological or chemical antifibrosis drugs. However, traditional Chinese medicines (TCMs) have been accumulated in this field for a long time and have advantages at present. Accordingly, many new TCM drugs against liver fibrosis, such as Fuzheng Huayu prescription (FZHY prescription), Dahuang Zhechong pill (DHZC), and Anluo Huaxian pill (ALHX), have been developed (Wei et al., 2015; Wang and Peng, 2018; Gui et al., 2020; Wu et al., 2020). These TCM drugs are all taken orally twice or three times a day. So far, there have been evidence-based medical research methods to evaluate the efficacy and safety of these TCM drugs combined with current first-line drug ETV (Wei et al., 2015; Wang and Peng, 2018; Wang et al., 2018). Although these meta-analysis could prove that the application of TCM drugs combined with ETV could improve HBV sufferers’ liver function and liver fibrosis, the problem that which different treatment strategies should be chosen by clinicians need to be solved. As is well known, network meta-analysis (NMA) can compare and rank the efficacy and safety of different TCM drugs, which can help clinicians write an appropriate prescription. Moreover, a relevant publication has been published using an NMA to evaluate the efficacy of eight TCM drugs for HBV-related liver fibrosis (Wang et al., 2020). However, the conclusion that FZHY prescription combined with ETV is the best therapeutic strategy for HBV liver fibrosis is very broad to subdivide clinical efficacy, indexes of liver fibrosis and liver function, TCM symptoms, and imaging indexes. As for improving the HBV-DNA negative conversion rates, no TCM strategies showed absolute superiority because of insufficient articles. Recently, there are some new relevant publications published in databases. Considering these factors, we are about to conduct an NMA to evaluate the comparative effects and rankings of all known dominated TCM drugs on CHB liver fibrosis.

## Materials and methods

This NMA was performed following the Preferred Reporting Items for Systematic Review and Meta-Analysis (PRISRMA) statement (Liberati et al., 2009) (Supplementary Information S1).

### Data sources and search strategy

We retrieved electronic databases of PubMed, MEDLINE Complete, OVID EMBASE, Scopus, Web of Science, Google Scholar, China National Knowledge Infrastructure (CNKI), and WanFang Data from their establishment to 17 Aug 2021. No language limitation was applied. The initial search strategies were performed as follows: “traditional Chinese medicine (TCM),” “Chinese medicine,” “herbs,” “herbal medicine,” “Fuzheng Huayu,” “Dahuang Zhechong,” “Anluo Huaxian,” “Biejia Ruangan,” “antiviral drugs/agents,” “entecavir,” “adefovir,” “chronic hepatits B liver fibrosis,” “chronic hepatits B,” “hepatitis B virus,” “liver fibrosis,” “hepatic fibrosis,” and “randomized controlled trials (RCTs).” Detailed information of all electronic databases is displayed in Supplementary Information S2.

### Inclusion and exclusion criteria

Two investigators (Yun-kai Dai and Hai-na Fan) independently read the abstracts and full articles. Following the criteria (participants, interventions, comparisons, outcomes, and study design, PICOS), we included certain items in this research: RCTs; TCMs or nucleos (t) ide analogs in interventions; adults only; course of treatment >1 month; and Jadad score >2. Meanwhile, some items should be excluded: HBV negative; decompensated liver cirrhosis; no serum indicator of liver fibrosis in outcomes; meta-analyses or systematic reviews; conference summaries or abstracts only; case reports; single-sex researches; animal experiments or fundamental experiment studies; incomplete or error information; scientific and technological achievements; non-pharmaceutical therapy; and duplicates.

### Data abstraction and quality evaluation

Two people (Yun-kai Dai and Yong-hong Hu), respectively, extracted relevant data and evaluated the methodological quality. Detailed information to be abstracted were listed as follows: first author and year of publication, patients’ age and gender, severity of the disease, courses of disease and treatment, interventions and outcomes (clinical efficacy, serum biomarkers of liver fibrosis, and serum parameters for liver function), administration route, and side effects. Missing information was remedied by getting in touch with the first or corresponding authors. Methodological quality evaluation of each literature was evaluated by means of the Cochrane Collaboration Recommendations assessment tool (Higgins et al., 2011). The assessment of risk of bias includes six domains: random sequence generation, allocation concealment, blinding of participants and personnel, blinding of outcomes assessment, incomplete outcome data, and selective reporting. Each element of these domains was used to evaluate the included trials as low, unclear, or high risk. Meanwhile, the Grading of Recommendations Assessment, Development, and Evaluation (GRADE), online guideline “https://gdt.gradepro.org/app/”, was applied to assess the evidence quality as high, moderate, low, and very low (Puhan et al., 2014).

### Statistical analysis

Stata version 13.0 software was used to compare the efficacy and safety of different TCM drugs across the included studies. Based on the Bayesian framework and the Markov chain Monte Carlo (MCMC) method, WinBUGS version 1.4.3 was used for the evaluation and procession of research data *a priori*. In order to accommodate the model, three chains and non-informative uniform and normal priori distributions were applied (Ades et al., 2006; Sutton et al., 2008). After that, to gain their posterior distributions, 10 thinning intervals each chain and 50,000 iterations were all set. As for the simulation iterations, the top 20,000 were used for annealing in order to eliminate the impacts of the initial value, while the bottom 30,000 were applied to sampling. As for effect sizes, the standardized mean difference (SMD) was produced for continuous variable data, and the relative risk (RR) was pooled for dichotomous outcomes. Both of them, conducting a random effects model to minimize the risk, were used for the summarization of each comparison effect, with their corresponding 95% confidence intervals (CIs), and a network plot, where node sizes are representative of the number of sufferers, while connection sizes are related to the number of RCTs, was produced to examine the direct and indirect evidence involving in multiple-intervention comparisons. The Brooks–Gelman–Rubin statistic using the potential scale reduction factor (PSRF) value was conducted to assess model convergence. Meanwhile, the node-splitting analysis was calculated to evaluate the consistency. The inconsistency index statistic (*I* ^*2*^) was used to quantify the heterogeneity between different treatment strategies. In order to investigate the stability of results, a sensitivity analysis was carried out. In addition, the surface under the cumulative ranking curve (SUCRA) was ranked to examine the efficacy and safety of all included strategies in each outcome.

## Results

### Literature screening

**FIGURE 1:**
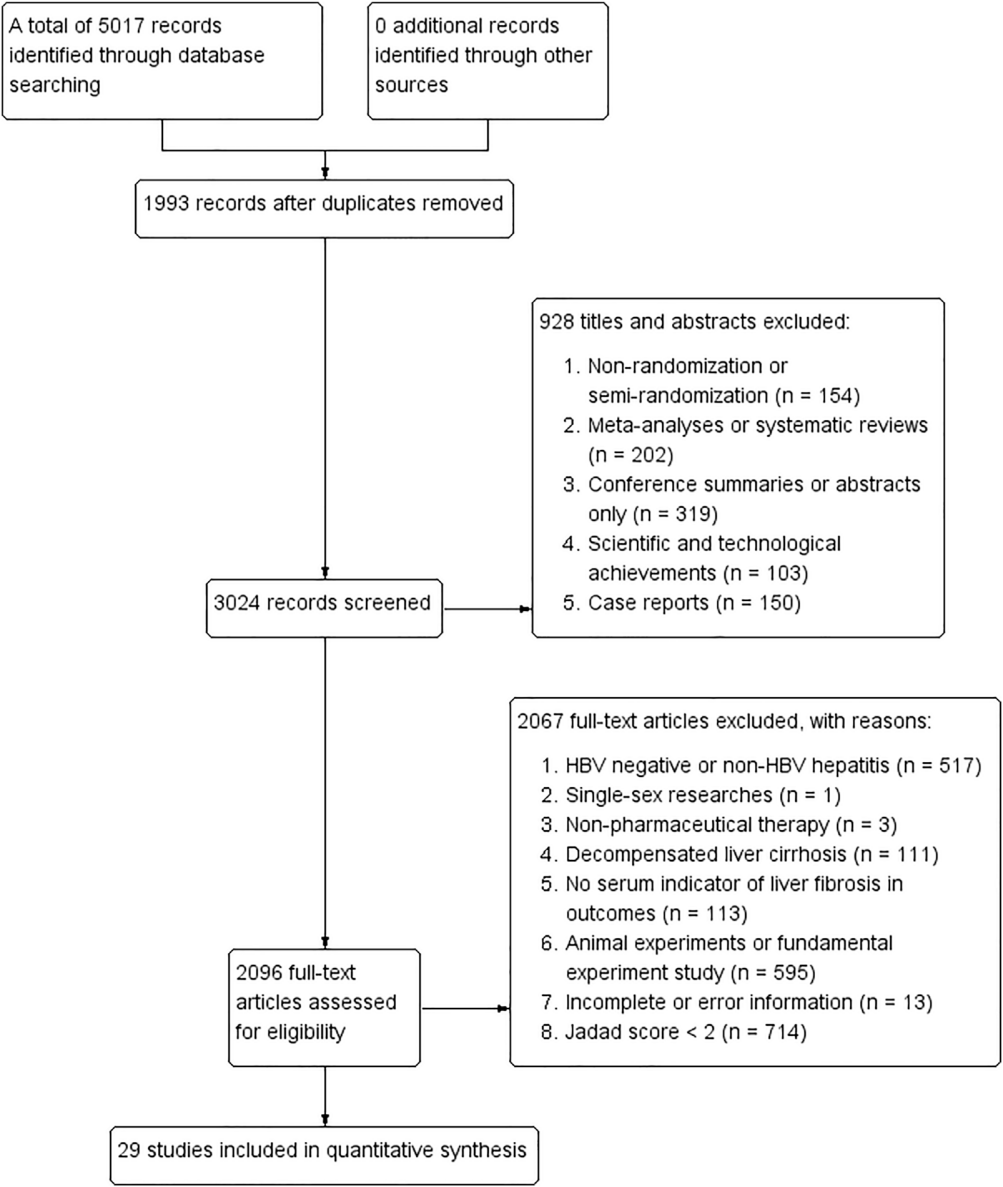
Flow diagram png.

Following the inclusion and exclusion criteria in search strategies, a total of 5,017 publications were identified using five databases, of which 1,993 records were removed because of duplicates, 928 were excluded by browsing titles and abstracts, and 2,067 were removed by reading full-text articles. Finally, 29 articles were included in this NMA (Wang et al., 2004; Wang, 2005; Zhang et al., 2006; Wang et al., 2010; Zhang et al., 2016; Gu, 2018; Li, 2018; Xiao and Hu, 2018; Xu et al., 2018; Zhang et al., 2018; Zhao, 2018; Chen et al., 2019; Liang, 2019; Shi, 2019; Di and Xia, 2020; Li et al., 2020; Rong et al., 2020; Wang, 2020; Yin et al., 2020; Yu et al., 2020; Zhang, 2020; Zhang and Li, 2020; Du et al., 2021; Huang, 2021; Li et al., 2021; Wang, 2021; Zhang, 2021; Zhao, 2021; Zhu et al., 2021). Detailed information of study selection can be found in Figure 1, and characteristics of the included RCTs are concluded in Table 1. Ingredients of each formula and quality control measures in all included publications are shown in Table 2. Accordingly, the composition of the four representative formulas including “Fuzheng Huayu tablet/capsule,” “Dahuang Zhechong pill,” “Anluo Huaxian pill,” and “Biejia Ruangan tablet” is listed in Supplementary Information S3.

**TABLE 1:** Characteristics of the studies included in this NMA.

Study	TCM syndrome	Course of disease (years)	Treatment group	Control group	Method of administration	Treatment course	Age (years)	Gender (male/female)	Outcome
(Huang, 2021)	N.R	T: 6.38 ± 1.19 C: 6.46 ± 1.28	HXHY + entecavir	Entecavir	Po. HXHY: b.i.d. Entecavir: qd	3 months	30–70	T: 13/12 C: 15/10	**B, C,**and **P**
(Zhao, 2021)	N.R	T: 5.04 ± 1.31 C: 4.76 ± 1.27	RJSJ + entecavir	Entecavir	Po. RJSJ: t.i.d. Entecavir: qd	3 months	36–64	T: 22/18 C: 24/16	**A, B, L,** and **N**
(Wang, 2021)	N.R	T: 5.51 ± 1.47 C: 5.28 ± 1.16	RJSJ + entecavir	Entecavir	Po. RJSJ: t.i.d. Entecavir: qd	6 months	20–76	T: 19/14 C: 18/15	**A, B,** and **C**
(Zhu et al., 2021)	N.R	T: 3.41 ± 0.74 C: 3.48 ± 0.70	RJSJ + entecavir	Entecavir	Po. RJSJ: b.i.d. Entecavir: qd	6 months	T: 49.84 ± 7.61 C: 49.21 ± 8.20	T: 20/16 C: 22/14	**A, B, C, D, K,** and **P**
(Du et al., 2021)	LKYD	N.R	ZYSG + entecavir	Entecavir	Po. ZYSG: b.i.d. Entecavir: qd	6 months	T: 45.1 ± 3.1 C: 50.1 ± 4.2	T: 18/12 C: 16/14	**A, B, C, E, F, G,** and **L**
(Li et al., 2021)	SQDBS	T: 5.43 ± 1.03 C: 5.45 ± 1.04	FZHY + entecavir	Entecavir	Po. FZHY: b.i.d. Entecavir: qd	36 weeks	T: 45.71 ± 5.03 C: 46.01 ± 5.24	T: 22/18 C: 24/16	**A, B, C, G, L, P,** and **Q**
(Zhang, 2021)	SLDSD and BSHMS	T: 15.83 ± 7.39 C: 15.21 ± 6.36	RJSJ + entecavir	Entecavir	Po. RJSJ: b.i.d. Entecavir: qd	12 months	26–69	T: 26/22 C: 25/23	**A, B, C,** and **K**
(Li et al., 2020)	N.R	T: 2.70 ± 1.50 C: 2.50 ± 1.40	FZHY + entecavir	Entecavir	Po. FZHY: t.i.d. Entecavir: qd	6 months	27–70	T: 36/15 C: 35/16	**A, B, C, G, H, J, K,** and **P**
(Yu et al., 2020)	N.R	T: 5.37 ± 2.74 C: 5.48 ± 2.28	RJSJ + entecavir	Entecavir	Po. RJSJ: b.i.d. Entecavir: qd	6 months	26–67	T: 23/17 C: 22/18	**A, B, C, G,** and **J**
(Wang, 2020)	DLS	T: 2.28 ± 0.83 C: 2.33 ± 0.71	RJSJ + entecavir	Entecavir	Po. RJSJ: b.i.d. Entecavir: qd	6 months	33–68	T: 28/27 C: 31/24	**A, B, C, J, L,** and **R**
(Rong et al., 2020)	N.R	N.R	RJSJ + entecavir	Entecavir	Po. RJSJ: b.i.d. Entecavir: qd	72 weeks	18–65	T: 243/115 C: 241/106	**A, D, J,** and **S**
(Di and Xia, 2020)	N.R	T: 8.67 ± 0.90 C: 8.89 ± 0.93	SGHY + entecavir	Entecavir	Po. SGHY: t.i.d. Entecavir: qd	6 months	34–69	T: 31/25 C: 33/23	**A, B, C, H, K,** and **G**
(Yin et al., 2020)	SLDSD	T: 17.08 ± 6.33 C: 16.32 ± 7.78	ZYSG + entecavir	Entecavir	Po. ZYSG: t.i.d. Entecavir: qd	6 months	18–70	T: 21/16 C: 24/14	**A, B, C, G, H, K, O, P,** and **T**
(Zhang and Li, 2020)	SBS	T: 13.60 C: 14.20	FZHY	RJSJ	Po. FZHY: t.i.d RJSJ: b.i.d	6 months	19–69	T: 33/7 C: 33/7	**B and H**
(Zhang, 2020)	N.R	T: 6.22 ± 1.27 C: 6.24 ± 1.31	ZYSG + entecavir	Entecavir	Po. ZYSG: b.i.d. Entecavir: qd	3 months	27–65	T: 24/16 C: 25/15	**A, B, C,** and **K**
(Liang, 2019)	N.R	T: 6.88 ± 2.05 C: 6.86 ± 2.01	FZHY + entecavir	Entecavir	Po. FZHY: t.i.d. Entecavir: qd	48 weeks	25–68	T: 28/20 C: 29/19	**B, C,** and **G**
(Chen et al., 2019)	N.R	N.R	FZHY + entecavir	Entecavir	Po. FZHY: t.i.d. Entecavir: qd	48 weeks	18.40–59.20	T: 50/14 C: 48/15	**B, C, L, O,** and **U**
(Shi, 2019)	N.R	T: 7.87 ± 1.32 C: 8.09 ± 1.44	SGHY + entecavir	Entecavir	Po. SGHY: b.i.d. Entecavir: qd	6 months	19–57	T: 25/16 C: 23/18	**A, B, C, L,** and **N**
(Li, 2018)	SBS	T: 5.31 ± 1.34 C: 5.45 ± 1.32	RJSJ + entecavir	Entecavir	Po. RJSJ: t.i.d. Entecavir: qd	12 months	T: 38.72 ± 3.27 C: 38.94 ± 3.32	T: 39/35 C: 42/32	**B, C, L,** and **N**
(Gu, 2018)	N.R	T: 22.18 ± 2.44 C: 22.26 ± 2.37 (months)	HXHY + RJSJ	RJSJ	Po. HXHY: b.i.d. RJSJ: t.i.d	6 months	T: 58.28 ± 13.17 C: 58.52 ± 13.28	T: 14/16 C: 13/17	**B, C,** and **G**
(Xiao and Hu, 2018)	SBS	N.R	FZHY + entecavir	Entecavir	Po. FZHY: b.i.d. Entecavir: qd	48 weeks	T: 27–64 C: 23–63	T: 18/7 C: 17/6	**B, C, H,** and **L**
(Zhao, 2018)	SBS	T: 6.41 ± 2.34 C: 6.33 ± 2.22	HXHY	RJSJ	Po. HXHY: b.i.d. RJSJ: t.i.d	24 weeks	T: 63.89 ± 3.28 C: 63.15 ± 3.33	T: 29/21 C: 30/20	**A** and **B**
(Zhang et al., 2018)	SLDSD	T: 27.40 ± 7.90 C: 28.20 ± 8.30 (months)	ZYSG	Entecavir	Po. ZYSG: qd. Entecavir: qd	24 weeks	T: 43.08 ± 8.49 C: 41.97 ± 7.84	T: 19/16 C: 17/18	**B, C, K, P, V,** and **W**
(Xu et al., 2018)	N.R	T: 6.52 ± 1.21 C: 6.26 ± 1.32	ZYSG + entecavir	Entecavir	Po. ZYSG: b.i.d. Entecavir: qd	12 months	T: 35.21 ± 12.43 C: 34.85 ± 11.47	T: 24/18 C: 23/19	**B, H, K, M, O,** and **P**
(Zhang et al., 2016)	SLDSD; SBS	T: 12.51 ± 4.36 C: 11.94 ± 6.85	RJSJ	Entecavir	Po. RJSJ: b.i.d. Entecavir: qd	24 weeks	18–65	T: 31/19 C: 29/21	**A, B, C, F, G, H, M,** and **P**
(Wang et al., 2010)	SQSBS	N.R	FZHY + RJSJ mimetics	RJSJ + FZHY mimetics	Po. FZHY: b.i.d. RJSJ: t.i.d	6 months	T: 41 C: 42	T: 53/19 C: 18/6	**B, M, P,** and **Y**
(Zhang et al., 2006)	SQDBS	2–17	FZHY	PYXZ	Po. FZHY: b.i.d. PYXZ: b.i.d	3 months	T: 44.30 C: 42.50	T: 35/25 C: 33/27	**A, B, C, G, H, I,** and **K**
(Wang, 2005)	N.R	T: 3.42 ± 2.75 C: 3.71 ± 2.91	RJSJ	PYXZ	Po. RJSJ: qd. PYXZ: t.i.d	3 months	T: 38.42 ± 12.13 C: 40.26 ± 15.11	T: 49/38 C: 20/16	**A, B,** and **C**
(Wang et al., 2004)	SQSBS	N.R	FZHY	RJSJ	Po. FZHY: b.i.d. RJSJ: t.i.d	6 months	T: 38.24 ± 11.33 C: 36.91 ± 10.38	T: 16/5 C: 17/5	**B, L,** and **M**

**Annotation:** N.R, not reported; SQDBS, syndrome of qi deficiency with blood stasis; LKYDS, liver–kidney yin deficiency syndrome; SLDSD, syndrome of liver depression and spleen deficiency; BSHMS, blood stasis and heat mutual syndrome; DLS, deficiency of liver and spleen; SBS, stasis block syndrome; SQSBS, syndrome of qi stagnation and blood stasis; SQDBS, syndrome of qi deficiency with blood stasis. T, treatment group. C, control group. HXHY, Huoxue Huayu therapy. RJSJ, Ruanjian Sanjie therapy. FZHY, Fuzheng Huayu therapy. ZYSG, Ziyin Shugan therapy. SGHY, Shugan Huayu therapy. PYXZ, Poyu Xiaozheng therapy. [qd], quaque die. [b.i.d], bis in die. [t.i.d], ter in die. **A**, clinical efficacy. **B**, serum biomarkers of live fibrosis indexes of hepatic fibrosis (including hyaluronic acid (HA), laminin (LN), pro-collagen type III (PC-III), or collagen-IV (IV-C)). **C**, serum parameters for liver function (including alanine aminotransferase (ALT), aspartate aminotransferase (AST), albumin (ALB), or total bilirubin (TBil)). **D**, Ishak fibrosis scores. **E**, fibrosis index based on the 4 factor (FIB-4). **F**, aspartate aminotransferase-to-platelet ratio index (APRI). **G**, hepatitis B virus-deoxyribonucleic acid (HBV-DNA) conversion rate and the quantification of HBV-DNA. **H**, imaging indexes (including thickness of the spleen, length of the spleen, the width of the portal vein, or the liver hardness). **I**, liver oblique diameter. **J**, liver stiffness in FibroScan before and after treatment. **K**, TCM, symptom scores (including hypochondriac pain, fatigue, abdominal distension, poor appetite, nausea and vomiting, xerostomia, choleplania, gloomy complexion, liver palms, depression, dark urine, yellow coating, stomach distension, physical impairment, insomnia and dreaminess, loose stool, dark tongue, laziness to speak, or hepatosplenomegaly). **L**, total TCM, symptom scores. **M**, efficacy of TCM, symptom scores. **N**, levels of inflammatory factors (tumor necrosis factor-α (TNF-α), interleukin-6 (IL-6), endothelin). **O**, immune function indexes (NK, CD3^+^, CD4^+^, CD8^+^, or CD4+/CD8+). **P**, adverse reaction. **Q**, chitinase 3 like protein 1 (CHI3L1). **R**, oxidative stress indicators (including superoxide dismutase (SOD) and malondialdehyde (MDA)). **S**, Knodell necroinflammatory score. **T**, coagulation function (prothrombin time, activated partial thromboplastin time, thrombin time, and fibrinogen). **U**, high-sensitivity C-reactive protein (hs-CRP). **V**, levels of Th1, Th2, Th17, and Treg cell ratio. **W**, short form 36-item health survey. **X**, HBV, markers before and after treatment. **Y**, score of B ultrasound.

**TABLE 2:** Ingredients of each formula and quality control measures in the included publications.

Author	Formula name	Ingredients of each formula				Quality control reported (Y/N)	Chemical analysis reported (Y/N)
(Huang, 2021)	Gexia Zhuyu decoction	*Paeonia suffruticosa Andr.* (Mu Danpi) 30 g	*Cyperus rotundus L.* (Xiang Fu) 20 g	*Paeonia lactiflora Pall.* (Chi Shao) 20 g	*Carthamus tinctorius L.* (Hong Hua) 20 g	Y-National Medical Products Administration	N
		*Aaugellica sinensis (Oliv) Diels.* (Dang Gui) 20 g	*Ligusticum chuanxiong Hort.* (Chuan Xiong) 15 g	*Citrus aurantium L.* (Zhi Qiao) 15 g	*Corydalis yanhusuo W.T. Wang* (Yan Husuo) 15 g		
		*Lindera aggregata (Sims) Kosterm*. (Wu Yao) 15 g	*Prunus persica (L.) Batsch* (Tao Ren) 15 g	*Radix Glycyrrhizae preparata* (Gan Cao) 10 g	*Trogopterus xanthipes Milne-Edwards* (Wu Lingzhi) 10 g		
(Zhao, 2021)	Erjia Ruanjian capsule	Erjia Ruanjian capsule 6 g [*Trionyx sinensis Wiegmann* (vinegared Bie Jia); *Whitemania pigra Whitman* (Shui Zhi); and *Citrus reticulata Blanco* (Chen Pi)]				Y-National Medical Products Administration	N
(Wang, 2021)	Fufang Biejia Ruangan tablet	Fufang Biejia Ruangan tablet 6 g [*Trionyx sinensis Wiegmann* (Bie Jia); *Curcuma phaeocaulis Val.* (E Zhu); *Paeonia lactiflora Pall.* (Chi Shao); and *Aaugellica sinensis (Oliv) Diels.* (Dang Gui)]				Y-National Medical Products Administration	N
(Zhu et al., 2021)	Huanglian Wendan decoction	*Coptis chinensis Franch.* (Huang Lian) 5 g	*Poria cocos (Schw.) Wolf* (Fu Lin) 30 g	*Bambusa tuldoides Munro* (Zhu Ru) 15 g	*Citrus reticulata Blanco* (Chen Pi) 10 g	Y-National Medical Products Administration	N
		*Pinellia ternata (Thunb) Breit.* (Ban Xia) 5 g	*Citrus aurantium L.* (Zhi Shi) 5 g	*Zingiber officinale Rose* (Sheng Jiang) 5 g	*Radix Glycyrrhizae preparata* (Gan Cao) 5 g		
(Du et al., 2021)	YiGuan decoction	*Adenophora tetraphylla (Thunb.) Fisch.* (Nan Sha Shen) 15 g	*Rehmannia glutinosa Libosch.* (Sheng Dihuang) 10 g	*Ophiopogon japonicus (Thunb.) Ker-Gawl.* (Mai Dong) 10 g	*Aaugellica sinensis(Oliv) Diels.* (Dang Gui) 10 g	Y-National Medical Products Administration	N
		*Lycium barbarum L.* (Gou Qizi) 10 g	*Melia toosendan Sieb. Et Zucc.* (Chuan Lianzi) 10 g	*Curcuma wenyujin Y.H.Chen et C.Ling* (Yu Jin) 10 g	*Cynanchum otophyllum* (Bai Shao) 15 g		
(Li et al., 2021)	Huoxue Ruanjian Fuzheng granule	*Sparganium stoloniferum Buch.-Ham* (San Leng)	*Curcuma phaeocaulis Val.* (E Zhu)	*Panax notoginseng (Burk.) F. H. Chen* (San Qi)	*Trionyx sinensis Wiegmann* (vinegared Bie Jia)	Y-National Medical Products Administration	N
		*Salvia miltiorrhiza Bge.* (Dan Shen)	*E pimedium brevicornum Maxim.* (Yin Yanghuo)	*Astragalus membranaceus* (Huang Qi)	*Codonopsis pilosula (Franch.) Nannf.* (Dang Shen)		
		*Ligustrum lucidum Ait.* (Nv Zhenzi)	*Eclipta prostrata L.* (Mo Hanlian)	*Ganoderma lucidum (Leyss.ex Fr.) Karst.* (Ling Zhi)	*Stevia rebaudiana (Bertoni) Hemsl.* (Tian Yeju)		
(Zhang, 2021)	Anluo Huaxian pill	Anluo Huaxian pill 6 g [*Rehmannia glutinosa Libosch.* (Sheng Dihuang); *Panax notoginseng (Burk.) F. H. Chen* (San Qi); *Whitemania pigra Whitman* (Shui Zhi); *Bombyx mori Linnaeus.* (Jiang Can); *Pheretima aspergillum (E. Perrier)* (Di Long); *Atractylodes macrocephala Koidz.* (Bai Zhu); *Curcuma wenyujin Y.H.Chen et C.Ling* (Yu Jin); *Bos taurus domesticus Gmelin* (Niu Huang); *Arca subcrenata Lischke* (Wa Lengzi); *Paeonia suffruticosa Andr.* (Mu Danpi); *Rheum palmatum L* (Da Huang); *Hordeum vulgare L.* (Raw Mai Ya); *Gallus domesticus Brisson* (Ji Neijin); and *Bubalus bubalis Linnaeus* (Shui Niujiao)]				Y-National Medical Products Administration	N
(Li et al., 2020)	Peitu Huazheng decoction	*Astragalus membranaceus* (Huang Qi) 30 g	*Atractylodes macrocephala Koidz.* (Bai Zhu) 30 g	*Trionyx sinensis Wiegmann* (Bie Jia) 30 g	C*uscuta chinensis Lam*. (Tu Sizi) 30 g	Y-National Medical Products Administration	N
		*Spatholobus suberectus Dunn* (Ji Xueteng) 30 g	*Cynanchum otophyllum* (Bai Shao) 30 g	*Dioscorea opposita Thunb.* (Shan Yao) 30 g	*Aaugellica sinensis (Oliv) Diels.* (Dang Gui) 20 g		
		*Bupleurum chinensis DC.* (Chai Hu) 15 g	*Citrus reticulata Blanco* (Chen Pi) 12 g	*Pinellia ternata (Thunb) Breit.* (Ban Xia) 9 g	*Prunus persica (L.) Batsch* (Tao Ren) 9 g		
		*Eupolyphaga sinensis Walk.* (Tu Biechong) 9 g	*Fritillaria thunbergii Miq.* (Zhe Beimu) 9 g	*Sinapis alba L*. (Bai Jiezi) 6 g	*Radix Glycyrrhizae preparata* (Gan Cao) 6 g		
(Yu et al., 2020)	Anluo Huaxian pill	Anluo Huaxian pill 6 g [*Rehmannia glutinosa Libosch.* (Sheng Dihuang); *Panax notoginseng (Burk.) F. H. Chen* (San Qi); *Whitemania pigra Whitman* (Shui Zhi); *Bombyx mori Linnaeus.* (Jiang Can); *Pheretima aspergillum (E. Perrier)* (Di Long); *Atractylodes macrocephala Koidz.* (Bai Zhu); *Curcuma wenyujin Y.H.Chen et C.Ling* (Yu Jin); *Bos taurus domesticus Gmelin* (Niu Huang)*; Arca subcrenata Lischke* (Wa Lengzi); *Paeonia suffruticosa Andr.* (Mu Danpi); *Rheum palmatum L* (Da Huang); *Hordeum vulgare L.* (Raw Mai Ya); *Gallus domesticus Brisson* (Ji Neijin); and *Bubalus bubalis Linnaeus* (Shui Niujiao)]				Y-National Medical Products Administration	N
(Wang, 2020)	Anluo Huaxian pill	Anluo Huaxian pill 6 g [*Rehmannia glutinosa Libosch.* (Sheng Dihuang); *Panax notoginseng (Burk.) F. H. Chen* (San Qi); *Whitemania pigra Whitman* (Shui Zhi); *Bombyx mori Linnaeus.* (Jiang Can); *Pheretima aspergillum (E. Perrier)* (Di Long); *Atractylodes macrocephala Koidz.* (Bai Zhu); *Curcuma wenyujin Y.H.Chen et C.Ling* (Yu Jin); *Bos taurus domesticus Gmelin* (Niu Huang); *Arca subcrenata Lischke* (Wa Lengzi); *Paeonia suffruticosa Andr.* (Mu Danpi); *Rheum palmatum L* (Da Huang); *Hordeum vulgare L.* (Raw Mai Ya); *Gallus domesticus Brisson* (Ji Neijin); and *Bubalus bubalis Linnaeus* (Shui Niujiao)]				Y-National Medical Products Administration	N
(Rong et al., 2020)	Biejia Ruangan tablet	Biejia Ruangan tablet 6 g [*Trionyx sinensis Wiegmann* (Bie Jia); *Curcuma phaeocaulis Val.* (E Zhu); *Paeonia lactiflora Pall.* (Chi Shao); and *Aaugellica sinensis (Oliv) Diels.* (Dang Gui)]				Y-National Medical Products Administration	N
(Di and Xia, 2020)	Heluo Shugan tablet	Heluo Shugan tablet 2.1 g [*Atractylodes macrocephala Koidz.* (Bai Zhu); *Cynanchum otophyllum* (Bai Shao); *Sparganium stoloniferum Buch.-Ham* (San Leng); *Cyperus rotundus L.* (Xiang Fu); *Curcuma phaeocaulis Val.* (E Zhu); *Aaugellica sinensis (Oliv) Diels.* (Dang Gui); *Chaenomeles speciosa*(*Sweet*)*Nakai* (Mu Gua); *Rheum palmatum L* (Da Huang); *Carthamus tinctorius L.* (Hong Hua); *Trionyx sinensis Wiegmann* (Bie Jia); *Prunus persica (L.) Batsch* (Tao Ren); *Curcuma wenyujin Y.H.Chen et C.Ling* (Yu Jin); *Artemisia scoparia Waldst. Et Kit.* (Yin Chen); *Sargassum pallidum (Turn.) C. Ag.* (Hai Zao); *Laminaria japonica Aresch.* (Kun Bu); *Scrophularia ningpoensis Hemsl.* (Xuan Shen); *Rehmannia glutinosa (Gdertn) Iibosch.* (Shu Dihuang); *Polygonum cuspidatum Sieb. Et Zucc.* (Hu Zhang); *Eupolyphaga sinensis Walk.* (Tu Biechong); *Bupleurum chinensis DC.* (Chai Hu); *Polygonum multiflorum Thuna.* (He Shouwu); *Campsis grandiflora* (*Thunb.*)*K.Schum.* (Ling Xiaohua); dung beetle; *Trogopterus xanthipes Milne-Edwards* (Wu Lingzhi); *Glycine* max (L.) merr. (Hei Dou); and *Lobelia chinensis Lour.* (Ban Bianlian)]				Y-National Medical Products Administration	N
(Yin et al., 2020)	Shugan Jianpi decoction	*Bupleurum chinensis DC.* (Chai Hu) 9 g	*Astragalus membranaceus* (Huang Qi) 20 g	*Atractylodes macrocephala Koidz.* (Bai Zhu) 15 g	*Poria cocos (Schw.) Wolf* (Fu Lin) 10 g	Y-National Medical Products Administration	N
		*Magnolia officinals Rehd.et Wils.* (Hou Po) 10 g	*Citrus reticulata Blanco* (Chen Pi) 9 g	*Curcuma wenyujin Y.H.Chen et C.Ling* (Yu Jin) 10 g	*Citrus aurantium L.* (Zhi Qiao) 10 g		
		*Cynanchum otophyllum* (Bai Shao) 15 g	*Aaugellica sinensis (Oliv) Diels.* (Dang Gui) 6 g	*Salvia miltiorrhiza Bge.* (Dan Shen) 9 g	*Radix Glycyrrhizae preparata* (Gan Cao) 6 g		
(Zhang and Li, 2020)	Xiongqi granule	*Astragalus membranaceus* (Huang Qi) 30 g	*Ligusticum chuanxiong Hort.* (Chuan Xiong) 15 g	*Curcuma wenyujin Y.H.Chen et C.Ling* (Yu Jin) 15 g	*Whitemania pigra Whitman* (Shui Zhi) 3 g	Y-National Medical Products Administration	N
		*Paeonia lactiflora Pall.* (Chi Shao) 15 g	*Aaugellica sinensis (Oliv) Diels.* (Dang Gui) 15 g	*Salvia miltiorrhiza Bge.* (Dan Shen) 15 g	*Paeonia suffruticosa Andr.* (Mu Danpi) 10 g		
		*Bupleurum chinensis DC.* (Chai Hu) 10 g	*Citrus aurantium L.* (Zhi Shi) 12 g	*Radix Glycyrrhizae preparata* (Gan Cao) 6 g			
(Zhang, 2020)	Yangxue Rougan decoction	*Spatholobus suberectus Dunn* (Ji Xueteng) 30 g	*Rehmannia glutinosa (Gdertn) Iibosch.* (Shu Dihuang) 15 g	*Schisandra chinesis (Turcz.) Baill* (Wu Weizi) 15 g	*Aaugellica sinensis (Oliv) Diels.* (Dang Gui) 15 g	Y-National Medical Products Administration	N
		*Ligusticum chuanxiong Hort.* (Chuan Xiong) 10 g	*Ziziphus jujuba Mill. Var. Spinosa* (*Bunge*) *Hu ex H. F. Chou* (Suan Zaoren) 30 g	*Trionyx sinensis Wiegmann* (Bie Jia) 15 g	*Bupleurum chinensis DC.* (Chai Hu) 10 g		
		*Gallus domesticus Brisson* (Ji Neijin) 15 g	*Chaenomeles speciosa* (*Sweet*) *Nakai* (Mu Gua) 15 g	E*quus asinus L*. (E Jiao) 10 g	*Cynanchum otophyllum* (Bai Shao) 15 g		
		*Whitemania pigra Whitman* (Shui Zhi) 3 g					
(Liang, 2019)	Fuzheng Huayu capsule	Fuzheng Huayu capsule 4.5 g [*Salvia miltiorrhiza Bge.* (Dan Shen); *Cordyeps sinensis* (*Berk.*) *Sacc* (Dongchong Xiacao); *Batsch* (Tao Ren); *Pini Pollen* (Songhua Fen); *Gynostemma pentaphllam* (*Thunb.*)*Makino* (Jiao Gulan); and *Schisandra chinesis (Turcz.) Baill* (Wu Weizi)]				Y-National Medical Products Administration	N
(Chen et al., 2019)	Fuzheng Huayu capsule	Fuzheng Huayu capsule 4.5 g [*Salvia miltiorrhiza Bge.* (Dan Shen); *Cordyeps sinensis* (*Berk.*) *Sacc* (Dongchong Xiacao); *Batsch* (Tao Ren); *Pini Pollen* (Songhua Fen); *Gynostemma pentaphllam* (*Thunb.*)*Makino* (Jiao Gulan); and *Schisandra chinesis (Turcz.) Baill* (Wu Weizi)]				Y-National Medical Products Administration	N
(Shi, 2019)	Shugan Tongluo decoction	*Bupleurum chinensis DC.* (Chai Hu) 10 g	*Cyperus rotundus L.* (Xiang Fu) 7 g	*Curcuma wenyujin Y.H.Chen et C.Ling* (Yu Jin) 10 g	*Corydalis yanhusuo W.T. Wang* (Yan Husuo) 10 g	Y-National Medical Products Administration	N
		*Trionyx sinensis Wiegmann* (Bie Jia) 15 g	*Salvia miltiorrhiza Bge.* (Dan Shen) 20 g	*Cynanchum otophyllum* (Bai Shao) 10 g	*Melia toosendan Sieb. Et Zucc.* (Chuan Lianzi) 7 g		
		*Lindera aggregata (Sims) Kosterm.* (Wu Yao) 10 g	*Ligusticum chuanxiong Hort.* (Chuan Xiong) 10 g	*Aucklandia lappa Decne* (Mu Xiang) 7 g	*Citrus reticulata Blanco* (Chen Pi) 10 g		
		*Radix Glycyrrhizae preparata* (Gan Cao) 15 g					
(Li, 2018)	Fufang Biejia Ruangan tablet	Fufang Biejia Ruangan tablet 6 g [*Trionyx sinensis Wiegmann* (Bie Jia); *Placenta Hominis* (Zi Heche); *F. H. Chen* (San Qi); *Codonopsis pilosula (Franch.) Nannf.* (Dang Shen); *Curcuma phaeocaulis Val.* (E Zhu); *Aaugellica sinensis (Oliv) Diels.* (Dang Gui); *Paeonia lactiflora Pall.* (Chi Shao); *lsatis indigotica Fort.* (Ban Langen); *Astragalus membranaceus* (Huang Qi); *Forsythia suspensa (Thunb.) Vahl* (Lian Qiao); *Cordyeps sinensis (Berk.) Sacc* (Dongchong Xiacao)]				Y-National Medical Products Administration	N
(Gu, 2018)	Gexia Zhuyu decoction	*Batsch* (Tao Ren) 6 g	*Dryopteris crassirhizoma Nakai* (Guan Zhong) 10 g	*Paeonia suffruticosa Andr.* (Mu Danpi) 10 g	*Scutellaria baicalensis Georg* (Huang Qin) 10 g	Y-National Medical Products Administration	N
		*Paeonia lactiflora Pall.* (Chi Shao) 10 g	*Ligusticum chuanxiong Hort.* (Chuan Xiong) 10 g	*Bupleurum chinensis DC.* (Chai Hu) 10 g	*Aaugellica sinensis (Oliv) Diels.* (Dang Gui) 15 g		
		*Salvia miltiorrhiza Bge.* (Dan Shen) 20 g	*Astragalus membranaceus* (Huang Qi) 20 g				
(Xiao and Hu, 2018)	Huayu Yangggan decoction	*Salvia miltiorrhiza Bge.* (Dan Shen) 20 g	*Trionyx sinensis Wiegmann* (Bie Jia) 20 g	*Ostreagigas Thunberg* (Mu Li) 20 g	*Rehmannia glutinosa Libosch.* (Sheng Dihuang) 20 g	Y-National Medical Products Administration	N
		*Aaugellica sinensis (Oliv) Diels.* (Dang Gui) 15 g	*Cynanchum otophyllum* (Bai Shao) 15 g	*Batsch* (Tao Ren) 15 g	*Carthamus tinctorius L.* (Hong Hua) 15 g		
		*Ligusticum chuanxiong Hort.* (Chuan Xiong) 15 g	*Whitemania pigra Whitman* (Shui Zhi) 10 g				
(Zhao, 2018)	Gexia Zhuyu decoction	*Aaugellica sinensis (Oliv) Diels.* (Dang Gui) 12 g	*Paeonia suffruticosa Andr.* (Mu Danpi) 12 g	*Lindera aggregata (Sims) Kosterm.* (Wu Yao) 12 g	*Cyperus rotundus L.* (Xiang Fu) 9 g	Y-National Medical Products Administration	N
		*Radix Glycyrrhizae preparata* (Gan Cao) 9 g	*Codonopsis pilosula (Franch.) Nannf.* (Dang Shen) 9 g	*Batsch* (Tao Ren) 9 g	*Carthamus tinctorius L.* (Hong Hua) 9 g		
		*Ligusticum chuanxiong Hort.* (Chuan Xiong) 9 g	*Paeonia lactiflora Pall.* (Chi Shao) 6 g	*Corydalis yanhusuo W.T. Wang* (Yan Hu Suo) 6 g			
(Zhang et al., 2018)	Shugan Jianpi granule	Shugan Jianpi granule 6 g [*Atractylodes lancea (Thunb.) DC.* (Cang Zhu); *Coix lacryma-jobi L.var.ma-yuen (Roman.) Stapf* (Yi Yiren); *Citrus aurantium L.* (Zhi Shi); *Dioscorea opposita Thunb.* (Shan Yao); *Aucklandia lappa Decne* (Mu Xiang); *Poria cocos (Schw.) Wolf* (Fu Lin); *C. pinnatifida Bge.* (Shan Zha); *Atractylodes macrocephala Koidz.* (Bai Zhu); *myristica fragrans Houtt* (Rou Doukou); and *Bupleurum chinensis DC.* (Chai Hu)]				Y-National Medical Products Administration	N
(Xu et al., 2018)	Shugan Jianpi decoction	*Astragalus membranaceus* (Huang Qi) 30 g	*Codonopsis pilosula (Franch.) Nannf.* (Dang Shen) 15 g	*Atractylodes macrocephala Koidz.* (Bai Zhu) 12 g	*Coix lacryma-jobi L.var.ma-yuen (Roman.) Stapf* (Yi Yiren) 15 g	Y-National Medical Products Administration	N
		*Poria cocos (Schw.) Wolf* (Fu Lin) 12 g	*Bupleurum chinensis DC.* (Chai Hu) 12 g	*Aaugellica sinensis (Oliv) Diels.* (Dang Gui) 12 g	*Citrus aurantium L.* (Zhi Qiao) 9 g		
		*Amomum villosum Lour* (Sha Ren) 9 g	*Curcuma wenyujin Y.H.Chen et C.Ling* (Yu Jin) 9 g	*Cynanchum otophyllum* Bai Shao) 12 g	*Citrus reticulata Blanco* (Chen Pi) 12 g		
		*Coptis chinensis Franch.* (Huang Lian) 6 g	*Radix Glycyrrhizae preparata* (Gan Cao) 6 g				
(Zhang et al., 2016)	Ruangan Huaxian decoction	*Codonopsis pilosula (Franch.) Nannf.* (Dang Shen) 20 g	*Bupleurum chinensis DC.* (Chai Hu) 10 g	*Curcuma wenyujin Y.H.Chen et C.Ling* (Yu Jin) 10 g	*Trionyx sinensis Wiegmann* Bie Jia) 30 g	Y-National Medical Products Administration	N
		*Salvia miltiorrhiza Bge.* (Dan Shen) 20 g	*Solanum nigrum L.* (Long Kui) 10 g	*Oldenlandia diffusa (willd.) Roxb.* (Baihua Sheshe Cao) 15 g	*Gynostemma pentaphllam* (*Thunb.*)*Makino* (Jiao Gulan) 15 g		
		*F. H. Chen* (San Qi) 10 g	*Curcuma longa.L.* (Jiang Huang) 10 g	*Paeonia lactiflora Pall.* (Chi Shao) 15 g	*Radix Glycyrrhizae preparata* (Gan Cao) 6 g		
(Wang et al., 2010)	Jiuwei Rougan granule	Jiuwei Rougan granule 10 g *Phyllanthus urinaria L.* (Zhen Zhucao); *Salvia miltiorrhiza Bge.* (Dan Shen); *Aaugellica sinensis (Oliv) Diels.* (Dang Gui); *Carthamus tinctorius L.* (Hong Hua); *Cyperus rotundus L.* (Xiang Fu); *Astragalus membranaceus* (Huang Qi); *Cordyeps sinensis (Berk.) Sacc* (Dongchong Xiacao); *Spatholobus suberectus Dunn* (Ji Xueteng); and *Bupleurum chinensis DC.* (Chai Hu)]				Y-National Medical Products Administration	N
(Zhang et al., 2006)	Shenjia Ronggan pill	Shenjia Ronggan pill 18 g [*Astragalus membranaceus* (Huang Qi); *Trionyx sinensis Wiegmann* (Bie Jia); *Polygonum cuspidatum Sieb. Et Zucc.* (Hu Zhang); *Atractylodes macrocephala Koidz.* (Bai Zhu); *Salvia miltiorrhiza Bge.* (Dan Shen); *F. H. Chen* (San Qi); *Cynanchum otophyllum* (Bai Shao); *Ligustrum lucidum Ait.* (Nv Zhenzi); *Spatholobus suberectus Dunn* (Ji Xueteng); and *Bupleurum chinensis DC.* (Chai Hu)]				Y-National Medical Products Administration	N
(Wang, 2005)	Ruanggan Kangxian decoction	*Bupleurum chinensis DC.* (Chai Hu) 6 g	*Curcuma wenyujin Y.H.Chen et C.Ling* (Yu Jin) 10 g	*Gallus domesticus Brisson* (Ji Neijin) 12 g	*Amomum villosum Lour* (Sha Ren) 5 g	Y-National Medical Products Administration	N
		*Astragalus membranaceus* (Huang Qi) 15 g	*Codonopsis pilosula (Franch.) Nannf.* (Dang Shen) 12 g	*Atractylodes macrocephala Koidz.* (Bai Zhu) 12 g	*Poria cocos (Schw.) Wolf* (Fu Lin) 12 g		
		*Salvia miltiorrhiza Bge.* (Dan Shen) 15 g	*Aaugellica sinensis (Oliv) Diels.* (Dang Gui) 12 g	*Prunus persica (L.) Batsch* (Tao Ren) 9 g	*Trionyx sinensis Wiegmann* (Bie Jia) 15 g		
		*Carapax Testudinis* (Gui Ban) 15 g					
(Wang et al., 2004)	Jiuwei Rougan granule	Jiuwei Rougan granule 20 g [*Phyllanthus urinaria L.* (Zhen Zhucao); *Salvia miltiorrhiza Bge.* (Dan Shen); *Aaugellica sinensis(Oliv) Diels.* (Dang Gui); *Carthamus tinctorius L.* (Hong Hua); *Cyperus rotundus L.* (Xiang Fu); *Astragalus membranaceus* (Huang Qi); *Cordyeps sinensis (Berk.) Sacc* (Dongchong Xiacao); *Spatholobus suberectus Dunn* (Ji Xueteng); and *Bupleurum chinensis DC.* (Chai Hu)]				Y-National Medical Products Administration	N

**Annotation:** *Italics* = Latin name of traditional Chinese medicinal materials. Y, yes; N, no.

### Risk of bias evaluation

**FIGURE 2:**
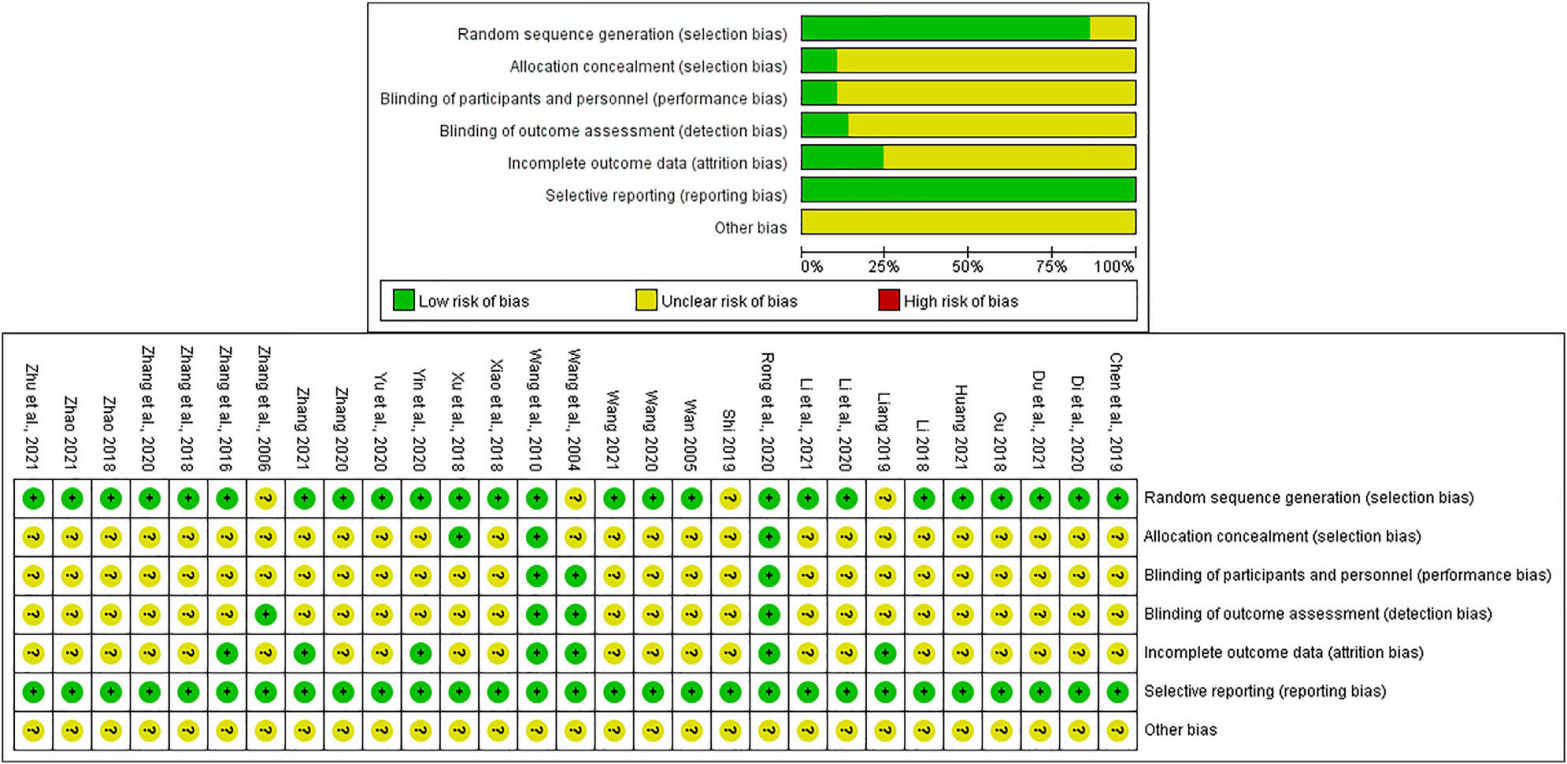
Risk of bias png.

We evaluated the risk of bias of each included publication’s quality with the aid of the Cochrane risk of bias assessment tool (Savovic et al., 2014). 1) Random sequence generation: 23 articles (Wang, 2005; Wang et al., 2010; Zhang et al., 2016; Gu, 2018; Li, 2018; Xiao and Hu, 2018; Zhang et al., 2018; Zhao, 2018; Chen et al., 2019; Di and Xia, 2020; Li et al., 2020; Rong et al., 2020; Wang, 2020; Yin et al., 2020; Yu et al., 2020; Zhang, 2020; Zhang and Li, 2020; Du et al., 2021; Huang, 2021; Li et al., 2021; Zhang, 2021; Zhao, 2021; Zhu et al., 2021) used random number table and two (Xu et al., 2018; Wang, 2021) used random envelope, which were viewed as “low risk”. However, the remaining four (Wang et al., 2004; Zhang et al., 2006; Liang, 2019; Shi, 2019) only reported “randomization,” so they were assessed as “unclear risk.” 2) Allocation concealment: only 3 RCTs (Wang et al., 2010; Xu et al., 2018; Rong et al., 2020) mentioned it while the rest did not. Therefore, the three trials were regarded as “low risk” while the rest as “unclear risk.” 3) Blinding (including participants and personnel, outcome evaluation): only four publications (Wang et al., 2004; Zhang et al., 2006; Wang et al., 2010; Rong et al., 2020) conducted the blinding method, and 25 (Wang, 2005; Zhang et al., 2016; Gu, 2018; Li, 2018; Xiao and Hu, 2018; Xu et al., 2018; Zhang et al., 2018; Zhao, 2018; Chen et al., 2019; Liang, 2019; Shi, 2019; Di and Xia, 2020; Li et al., 2020; Wang, 2020; Yin et al., 2020; Yu et al., 2020; Zhang, 2020; Zhang and Li, 2020; Du et al., 2021; Huang, 2021; Li et al., 2021; Wang, 2021; Zhang, 2021; Zhao, 2021; Zhu et al., 2021) had insufficient information. Therefore, they were successively estimated as “low risk” and “unclear risk.” 4) Incomplete outcome data: only seven trials (Wang et al., 2004; Wang et al., 2010; Zhang et al., 2016; Liang, 2019; Rong et al., 2020; Yin et al., 2020; Zhang, 2021) described the situation of withdrawal or dropout leading to “low risk,” while the rest did not mention it resulting in “unclear risk.” 5) Selective reporting: all of the included studies were regarded as “low risk” due to the acquirement of the complete implementation scheme. 6) Other bias: considering that some certain unknown or unexpected biases could potentially influence the results of this NMA, the 29 articles were evaluated as “unclear risk.” The aforementioned detailed risk of bias evaluation is displayed in Figure 2.

### Network evidence and analysis

**FIGURE 3:**
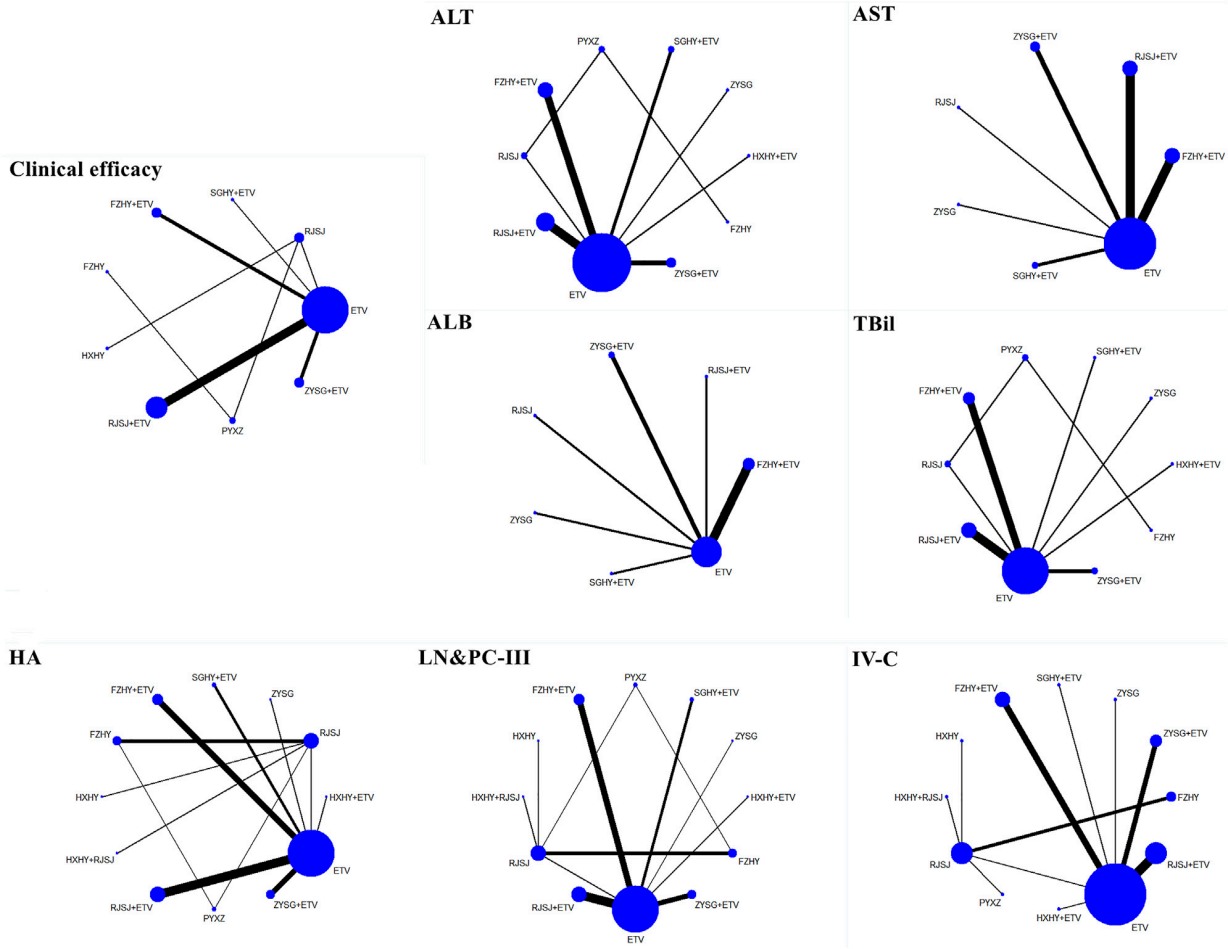
Network evidence of primary endpoints (clinical efficacy, indexes of hepatic fibrosis, and liver function) png.

**FIGURE 4:**
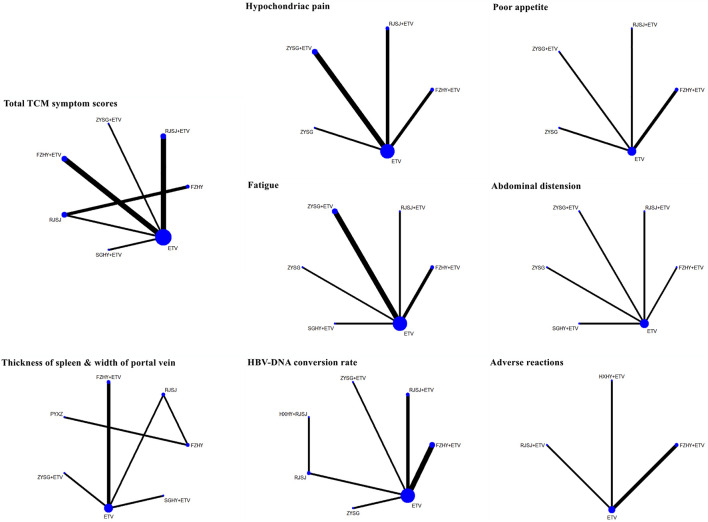
Network evidence of secondary endpoints (TCMs symptom scores, imaging indexes, HBV-DNA conversion rate, and adverse reactions) png.

A total of 13 regimens in this NMA were as follows: Fuzheng Huayu therapy (FZHY), Huoxue Huayu therapy (HXHY), Ruanjian Sanjie therapy (RJSJ), Ziyin Shugan therapy (ZYSG), Shugan Huayu therapy (SGHY), Poyu Xiaozheng therapy (PYXZ), FZHY + ETV, HXHY + ETV, RJSJ + ETV, ZYSG + ETV, SGHY + ETV, HXHY + RJSJ, and ETV. Briefly speaking, primary outcomes included clinical efficacy, indexes of hepatic fibrosis (hyaluronic acid (HA), laminin (LN), pro-collagen type III (PC-III), or collagen-IV (IV-C)), and indexes of liver function (alanine aminotransferase (ALT), aspartate aminotransferase (AST), albumin (ALB), or total bilirubin (TBil)). Secondary outcomes included total TCM symptom scores, TCM symptom scores (hypochondriac pain, fatigue, abdominal distension, or poor appetite), imaging indexes (thickness of spleen, length of spleen, width of portal vein, or the liver hardness), hepatitis B virus-deoxyribonucleic acid (HBV-DNA) conversion rate and the quantification of HBV-DNA, and rates of adverse reactions. Network evidence of all the outcomes can be found in Figure 3 (primary outcomes) and Figure 4 (secondary outcomes). As shown in Supplementary Information S4, results of node-splitting between direct and indirect evidence suggested consistency for the aforementioned primary and secondary endpoints (*p* > 0.05). Accordingly, the PSRF value with 1 or 1.01 proved no divergence and had a stable result. As a consequence, a consistent model was performed.

### Primary endpoints

#### Clinical efficacy

**FIGURE 5:**
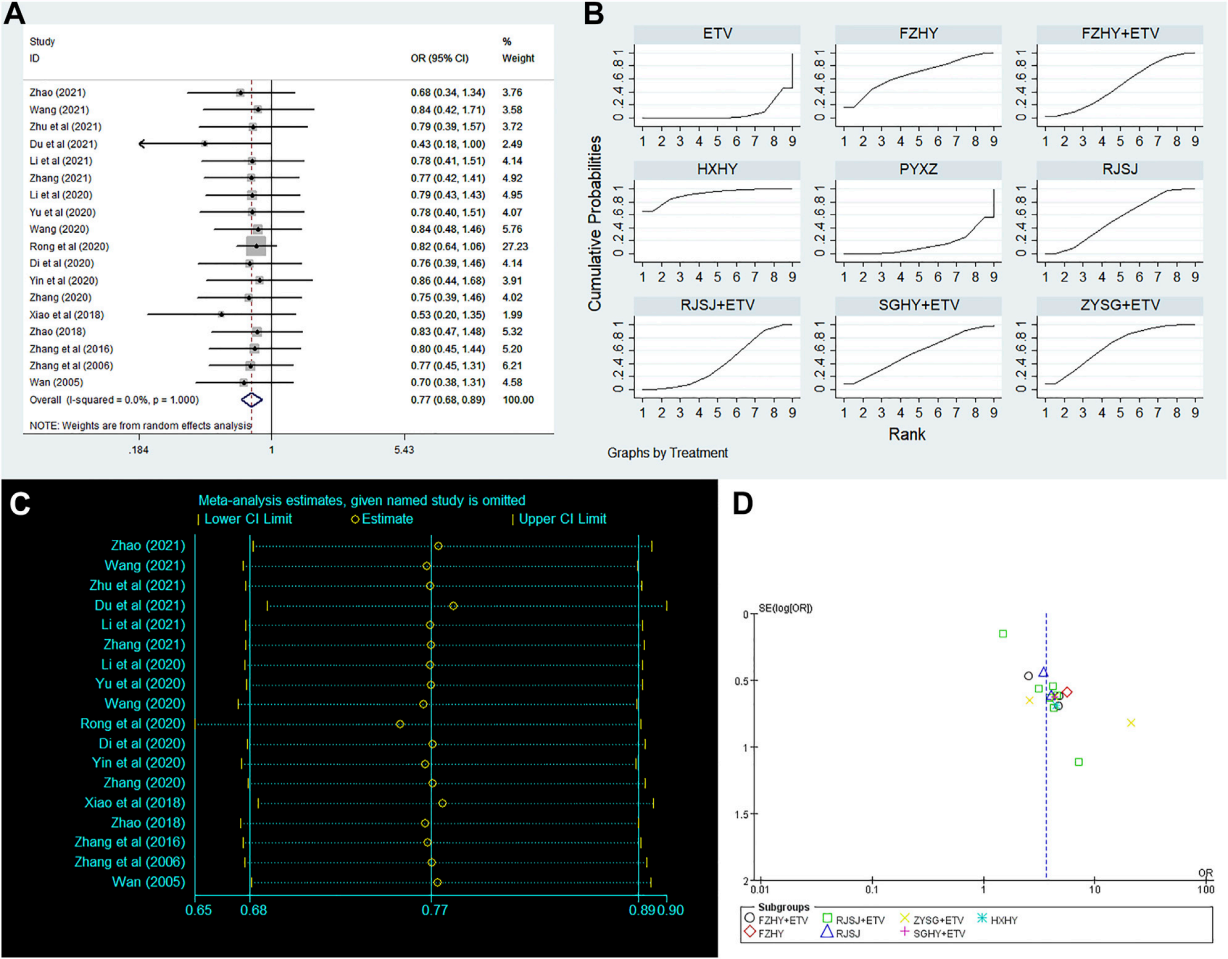
Clinical efficacy (**(A)** forest plot; **(B)** SUCRA plot; **(C)** sensitivity analysis; and **(D)** funnel plot) png.

Clinical efficacy of liver fibrosis was researched by 18 publications involving in nine interventions (ETV, RJSJ + ETV, FZHY + ETV, ZYSG + ETV, SGHY + ETV, FZHY, RJSJ, PYXZ, and HXHY) (Wang, 2005; Zhang et al., 2006; Zhang et al., 2016; Zhao, 2018; Shi, 2019; Di and Xia, 2020; Li et al., 2020; Rong et al., 2020; Wang, 2020; Yin et al., 2020; Yu et al., 2020; Zhang, 2020; Du et al., 2021; Li et al., 2021; Wang, 2021; Zhang, 2021; Zhao, 2021; Zhu et al., 2021). As shown in Figure 5A, result of heterogeneity analysis indicated good homogeneity (*p* = 0.18 > 0.05), and result of the SUCRA plot (Figure 5B) suggested HXHY (91.2%) ranked first, followed by FZHY (67.4%) and ZYSG + ETV (67.4%). In addition, strong stability in the ranking of the nine interventions was observed in the sensitivity analysis (Figure 5C), and the asymmetry funnel plot of clinical efficacy was exhibited in Figure 5D.

#### Serum biomarkers of liver fibrosis (HA, LN, PC-III, and IV-C)

**FIGURE 6:**
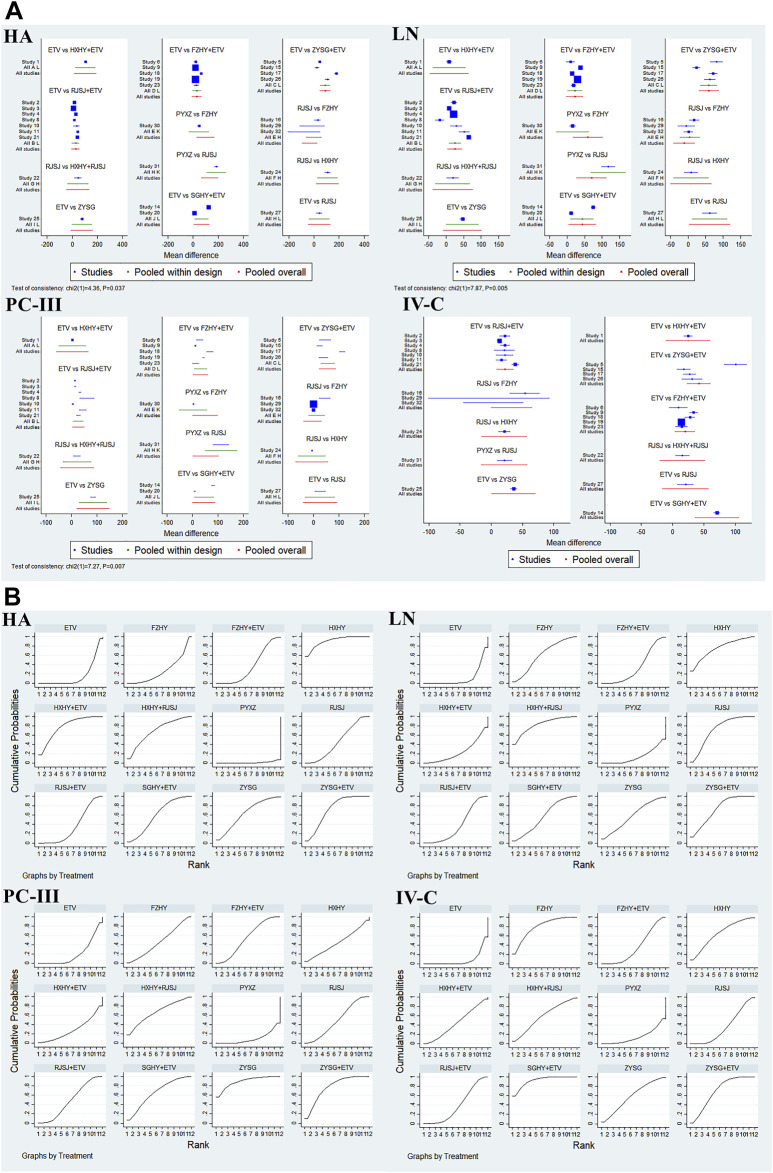
Four indexes of liver fibrosis (**(A)** test of consistency and **(B)** SUCRA value) png.

In this NMA, 28 RCTs with different treatments (FZHY + ETV, RJSJ + ETV, SGHY + ETV, HXHY + ETV, ZYSG + ETV, HXHY + RJSJ, FZHY, RJSJ, ZYSG, HXHY, PYXZ, and ETV) reported the HA, LN, and PC-III (Wang et al., 2004; Wang, 2005; Zhang et al., 2006; Wang et al., 2010; Zhang et al., 2016; Gu, 2018; Li, 2018; Xiao and Hu, 2018; Xu et al., 2018; Zhang et al., 2018; Zhao, 2018; Chen et al., 2019; Liang, 2019; Shi, 2019; Di and Xia, 2020; Li et al., 2020; Wang, 2020; Yin et al., 2020; Yu et al., 2020; Zhang, 2020; Zhang and Li, 2020; Du et al., 2021; Huang, 2021; Li et al., 2021; Wang, 2021; Zhang, 2021; Zhao, 2021; Zhu et al., 2021), and 26 with the same interventions described the IV-C (Wang et al., 2004; Wang, 2005; Wang et al., 2010; Zhang et al., 2016; Gu, 2018; Li, 2018; Xiao and Hu, 2018; Xu et al., 2018; Zhang et al., 2018; Zhao, 2018; Chen et al., 2019; Liang, 2019; Di and Xia, 2020; Li et al., 2020; Wang, 2020; Yin et al., 2020; Yu et al., 2020; Zhang, 2020; Zhang and Li, 2020; Du et al., 2021; Huang, 2021; Li et al., 2021; Wang, 2021; Zhang, 2021; Zhao, 2021; Zhu et al., 2021). Test of consistency including the four indexes of liver fibrosis showed good consistency (*p* < 0.05) (Figure 6A). As for the results of the SUCRA value (Figure 6B), the SUCRA plot of HA exhibited that FZHY (90.8%) ranked first, followed by HXHY + ETV (77.6%) and ZYSG + ETV (72.8%). The plot of LN indicated that HXHY + RJSJ (82.5%) ranked first, followed by HXHY (74.9%) and ZYSG + ETV (72.9%). The plot of PC-III suggested ZYSG (88.9%) ranked first, followed by ZYSG + ETV (77.0%) and HXHY + RJSJ (66.7%). The plot of IV-C showed that SGHY + ETV (92.0%) ranked first, followed by FZHY (79.0%) and ZYSG + ETV (69.6%).

#### Serum parameters for liver function (alanine aminotransferase, aspartate aminotransferase, albumin, and total bilirubin)

**FIGURE 7:**
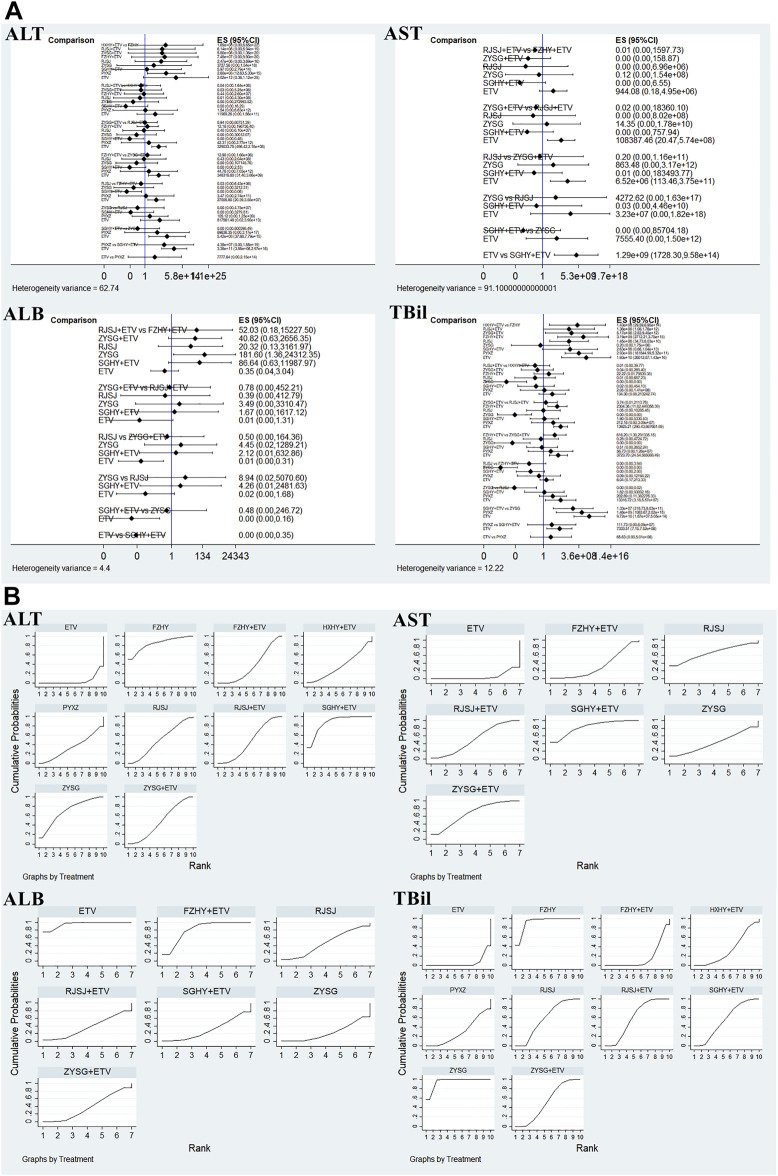
Four serum parameters for liver function (**(A)** test of heterogeneity and **(B)** SUCRA value) png.

In terms of the four serum parameters of liver function, 21 researches with 10 various therapies (FZHY + ETV, RJSJ + ETV, SGHY + ETV, HXHY + ETV, ZYSG + ETV, FZHY, RJSJ, ZYSG, PYXZ, and ETV) reported the ALT (Wang, 2005; Zhang et al., 2006; Zhang et al., 2016; Li, 2018; Xiao and Hu, 2018; Zhang et al., 2018; Chen et al., 2019; Liang, 2019; Shi, 2019; Di and Xia, 2020; Li et al., 2020; Wang, 2020; Yin et al., 2020; Yu et al., 2020; Zhang, 2020; Du et al., 2021; Huang, 2021; Li et al., 2021; Wang, 2021; Zhang, 2021; Zhu et al., 2021). A total of 17 trials with seven treatments (FZHY + ETV, RJSJ + ETV, ZYSG + ETV, SGHY + ETV, RJSJ, ZYSG, and ETV) reported the AST (Zhang et al., 2016; Li, 2018; Xiao and Hu, 2018; Zhang et al., 2018; Chen et al., 2019; Liang, 2019; Shi, 2019; Di and Xia, 2020; Li et al., 2020; Wang, 2020; Yin et al., 2020; Zhang, 2020; Du et al., 2021; Li et al., 2021; Wang, 2021; Zhang, 2021; Zhu et al., 2021). A total of 11 publications reported the ALB (Zhang et al., 2006; Zhang et al., 2016; Xiao and Hu, 2018; Zhang et al., 2018; Liang, 2019; Di and Xia, 2020; Li et al., 2020; Yin et al., 2020; Yu et al., 2020; Zhang, 2020; Li et al., 2021). However, because one article was different from the other 10 in terms of evaluation criterion (Zhang et al., 2006), the remaining 10 with the same treatments as AST were used for subsequent analysis. In addition, there were 17 RCTs with the same as ALT reported the TBil (Wang, 2005; Zhang et al., 2006; Zhang et al., 2016; Zhang et al., 2018; Chen et al., 2019; Liang, 2019; Di and Xia, 2020; Li et al., 2020; Wang, 2020; Yin et al., 2020; Yu et al., 2020; Zhang, 2020; Huang, 2021; Li et al., 2021; Wang, 2021; Zhang, 2021; Zhu et al., 2021). Test of heterogeneity including the four indexes of liver function showed good homogeneity (*p* > 0.05) (Figure 7A). As for the results of the SUCRA value (Figure 7B), the optimal value of ALT was SGHY + ETV (87.3%), second was FZHY (84.0%), and third was ZYSG (69.9%). The top three of AST about the value were SGHY + ETV (83.4%), ZYSG + ETV (67.7%), and RJSJ (66.9%) in order. The SUCRA plot of ALB suggested ETV (95.6%) ranked first, followed by FZHY + ETV (81.2%) and RJSJ (46.5%). As for the TBil, the highest SUCRA value was found for ZYSG (95.1%), second was FZHY (92.8%), and third was RJSJ + ETV (60.4%).

### Secondary endpoints

#### Traditional Chinese medicine symptom scores

**FIGURE 8:**
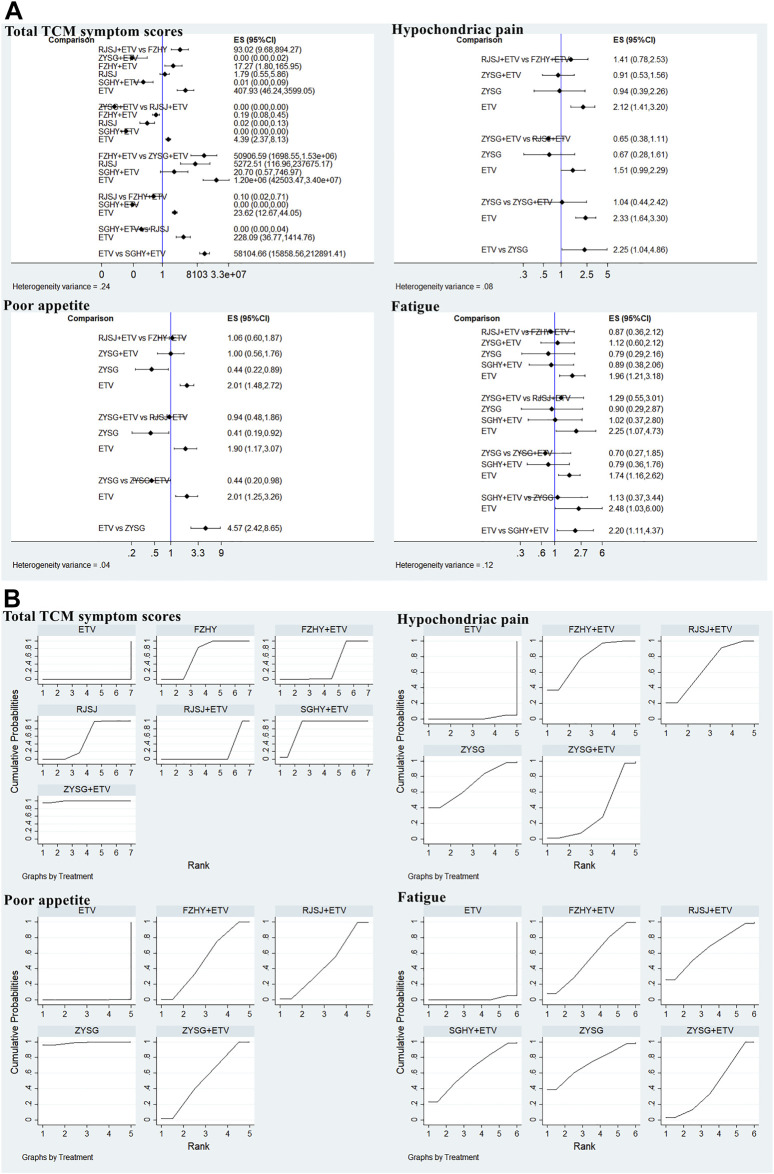
TCMs symptom scores (**(A)** test of heterogeneity and **(B)** SUCRA value) png.

In this study, 11 publications with different therapies (FZHY + ETV, RJSJ + ETV, ZYSG + ETV, SGHY + ETV, FZHY, RJSJ, and ETV) calculated the total TCM symptom scores (Wang et al., 2004; Wang et al., 2010; Zhang et al., 2016; Li, 2018; Xiao and Hu, 2018; Chen et al., 2019; Shi, 2019; Wang, 2020; Du et al., 2021; Li et al., 2021; Zhao, 2021). Considering one article having a discrepancy in evaluation criterion in this scores (Zhang et al., 2006), it was not used for subsequent analysis. As for the hypochondriac pain, eight trials with interventions (FZHY + ETV, RJSJ + ETV, ZYSG + ETV, ZYSG, and ETV) mentioned it (Xu et al., 2018; Zhang et al., 2018; Liang, 2019; Li et al., 2020; Yin et al., 2020; Zhang, 2020; Zhang, 2021; Zhu et al., 2021). Five with the same therapies as hypochondriac pain reported poor appetite (Zhang et al., 2018; Liang, 2019; Li et al., 2020; Yin et al., 2020; Zhang, 2021). Eight RCTs with six treatments (FZHY + ETV, RJSJ + ETV, ZYSG + ETV, SGHY + ETV, ZYSG, and ETV) mentioned fatigue (Xu et al., 2018; Zhang et al., 2018; Liang, 2019; Di and Xia, 2020; Li et al., 2020; Yin et al., 2020; Zhang, 2020; Zhang, 2021). Five articles with the same treatments as fatigue reported abdominal distension (Zhang et al., 2018; Liang, 2019; Di and Xia, 2020; Yin et al., 2020; Zhang, 2021). Test of heterogeneity including total TCM symptom scores, hypochondriac pain, and fatigue showed good homogeneity (*p* > 0.05) (Figure 8A). In terms of the SUCRA value (Figure 8B), the highest value of total TCM symptom scores was observed for ZYSG + ETV (99.2%), followed by SGHY + ETV (84.2%) and FZHY (63.8%). The top three of hypochondriac pain about the value were FZHY + ETV (78.1%), ZYSG (70.0%), and RJSJ + ETV (67.2%) in order. The optimal value of poor appetite was ZYSG (98.8%), followed by ZYSG + ETV (52.8%) and FZHY + ETV (52.2%). As for the fatigue, ZYSG accounting for 71.3% ranked first, RJSJ + ETV for 65.5% ranked second, and SGHY + ETV for 64.3% ranked third.

#### Imaging indexes

**FIGURE 9:**
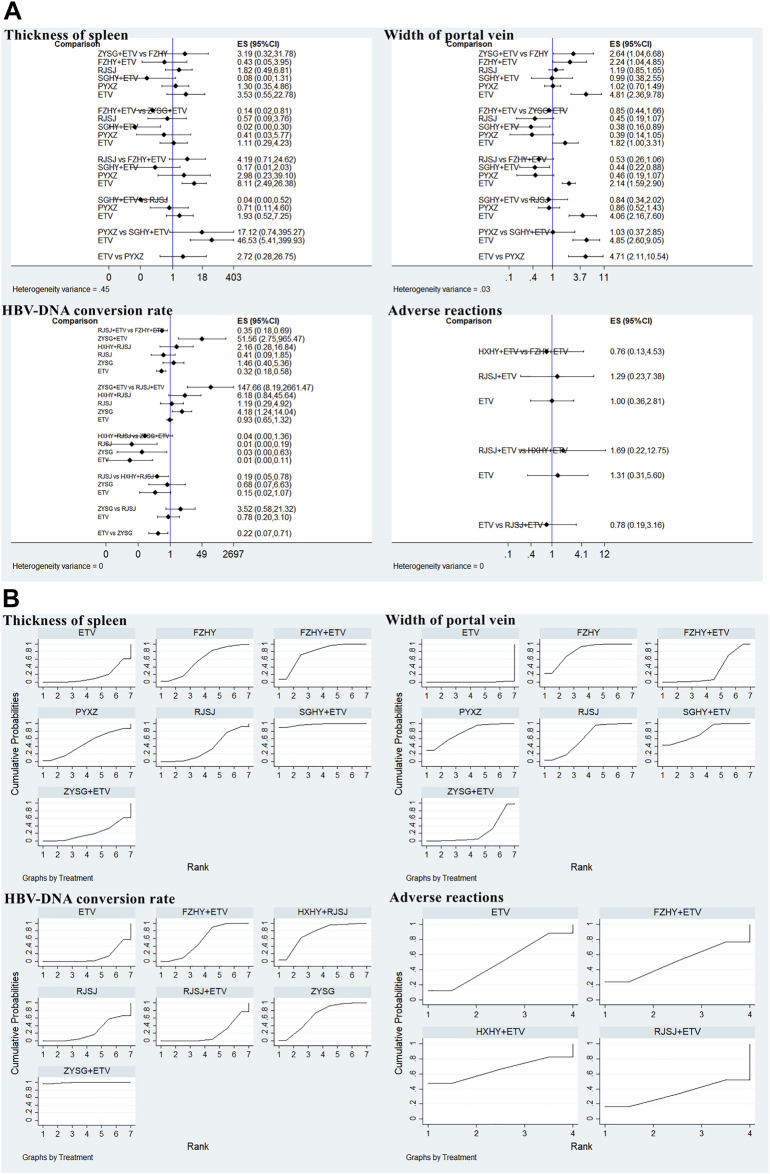
Two imaging indexes, HBV-DNA conversion rate, and adverse reactions (**(A)** test of heterogeneity and **(B)** SUCRA value) png.

Considering the included trials which reported the indexes of length of the spleen and liver hardness were inconsistent with each other in terms of evaluation criterion, so they were only qualitatively described. In this study, both thickness of the spleen and width of the portal vein were mentioned in seven included studies with seven therapies (FZHY + ETV, ZYSG + ETV, SGHY + ETV, FZHY, RJSJ, PYXZ, and ETV) (Zhang et al., 2006; Zhang et al., 2016; Xiao and Hu, 2018; Di and Xia, 2020; Li et al., 2020; Yin et al., 2020; Zhang and Li, 2020). Results of heterogeneity variance showed good homogeneity in thickness of the spleen (*p* > 0.05) except for the width of the portal vein (Figure 9A). As for the rankings of the SUCRA value (Figure 9B), result of thickness of the spleen showed that SGHY + ETV (97.3%) ranked first, FZHY + ETV (76.3%) was second, and FZHY (57.7%) was third. Result of the width of the portal vein exhibited that FZHY (80.4%) had the highest value, SGHY + ETV (77.6%) ranked second, and PYXZ (76.6%) ranked third.

#### Hepatitis B virus-deoxyribonucleic acid conversion rate

There were nine trials with seven treatments (FZHY + ETV, RJSJ + ETV, ZYSG + ETV, HXHY + ETV, RJSJ, ZYSG, and ETV), which reported this endpoint (Zhang et al., 2016; Gu, 2018; Zhang et al., 2018; Liang, 2019; Li et al., 2020; Rong et al., 2020; Yin et al., 2020; Yu et al., 2020; Li et al., 2021), and there were heterogeneity existed in the test of heterogeneity (*p* < 0.05) (Figure 9A). In terms of rankings, ZYSG + ETV (99.1%) had the highest SUCRA value, followed by HXHY + RJSJ (73.0%) and ZYSG (66.3%) (Figure 9B).

#### Adverse reactions

Four RCTS with four interventions (FZHY + ETV, RJSJ + ETV, HXHY + ETV, and ETV) mentioned adverse reactions (Li et al., 2020; Huang, 2021; Li et al., 2021; Zhu et al., 2021). Because heterogeneity variance was zero, heterogeneity was observed in this outcome (Figure 9A). Result of SUCRA rankings indicated that HXHY + ETV (65.4%) ranked first, followed by FZHY + ETV (50.3%) and ETV (50.2%) (Figure 9B).

### Other endpoints

In this NMA, Ishak fibrosis scores were reported by two trials with RJSJ + ETV (Rong et al., 2020; Zhu et al., 2021). Liver stiffness in FibroScan before and after treatment was reported by four RCTs with two treatments (FZHY + ETV and RJSJ + ETV) (Li et al., 2020; Rong et al., 2020; Wang, 2020; Yu et al., 2020). Fibrosis index based on the 4 factor (FIB-4) was mentioned by only one document with ZYSG + ETV (Du et al., 2021). Aspartate aminotransferase-to-platelet ratio index (APRI) was mentioned by two articles with two interventions (ZYSG + ETV and RJSJ) (Zhang et al., 2016; Du et al., 2021). Considering few publications and discrepancy in assessment criteria, these indexes of hepatic histopathology were only qualitatively described. But results showed whatever treatment strategies did patients choose, TCM drugs or combined with ETV were more effective than single usage of ETV.

### Rating evidence of quality by grading of recommendations assessment, development, and evaluation system

**FIGURE 10:**
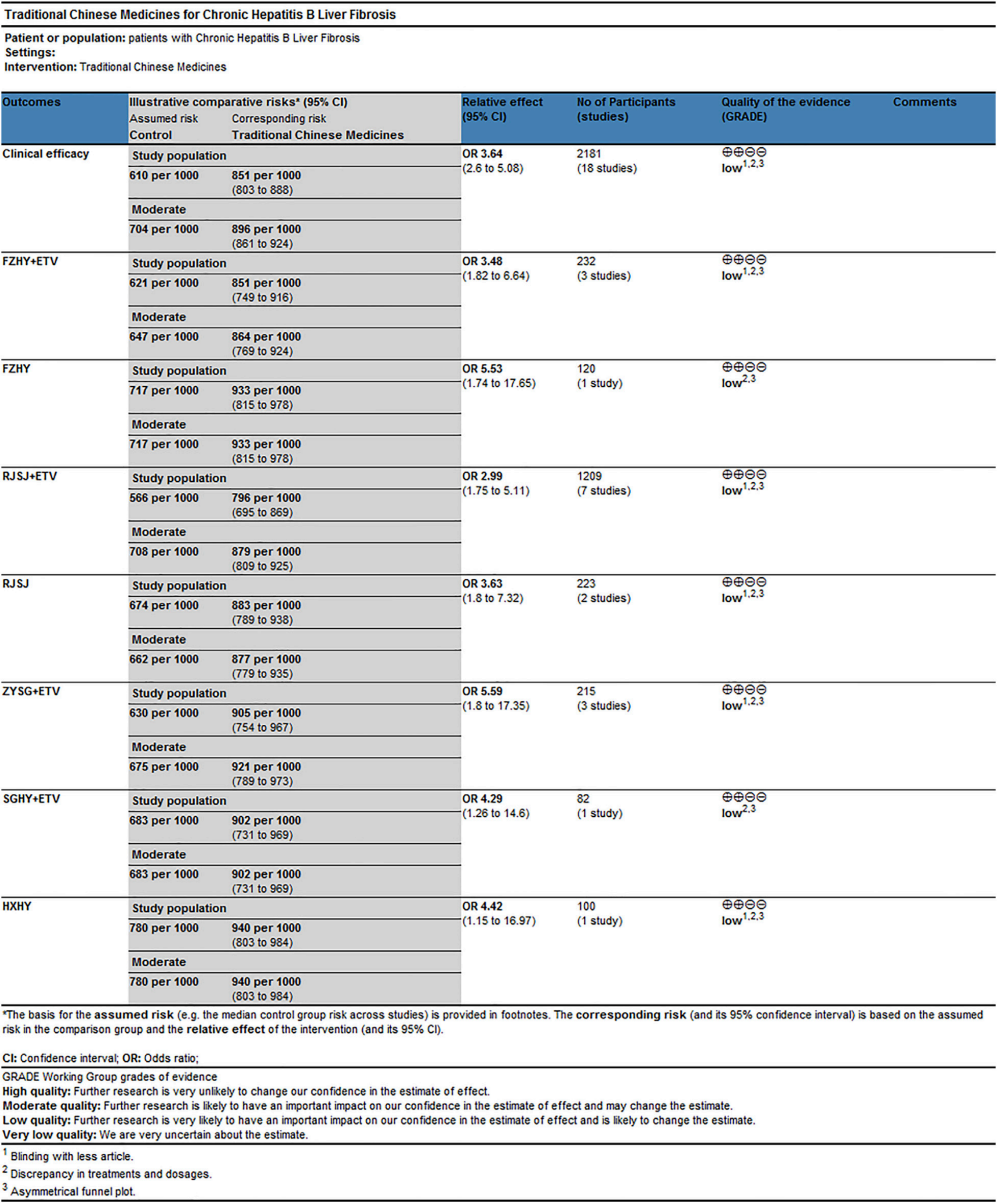
GRADE quality grading evaluation of clinical efficacy png.

As for the clinical efficacy, the reasons for downgrading or upgrading the quality of evidence were as follows (Figure 10): 1) blinding with fewer articles; 2) discrepancy in treatments and dosages; and 3) asymmetrical funnel plot. Result of quality of estimates showed “Low.” They could be associated with risk of bias and publication bias.

## Discussion

NMA allows the development of credible ranking systems of the likely efficacy and safety of various treatment strategies to help clinicians to make decisions even in the absence of head-to-head RCTs (Salanti et al., 2011). The liver biopsy is the gold standard for the diagnosis and prognosis of liver fibrosis. However, because of few RCTs involving in TCM treatments with description of Ishak fibrosis scores, qualitative description was conducted in this study. Nevertheless, our findings indicated that FZHY or combined with ETV might be a better choice to improve the clinical efficacy, recover serum biomarkers of liver fibrosis (including HA and IV-C), and serum parameters for liver function (ALT, ALB, and TBil), lowered the total TCM symptom scores, relieved hypochondriac pain and poor appetite, regained the width of portal vein and thickness of spleen, and reduced the occurrence of adverse reactions. In addition, ZYSG or combined with ETV could also recover the level of LN, PC-III, and AST and improve fatigue and the HBV-DNA conversion rate. As for the safety of TCM drugs, our results showed no serious adverse event during the period of treatment. TCM drugs or combined with ETV also suggested low risk of side effects. Therefore, their safety should be worth affirming.

Hepatic fibrosis is a key pathological process of chronic hepatitis to cirrhosis and presents in most chronic liver diseases. It can also cause structural and functional abnormalities of the liver, which seriously threatens the health of patients. Currently, HBV infection is still the main cause of chronic liver disease in China. HBV-mediated immune response leads to repeated liver cell damage and inflammatory response, promoting the progression of cirrhosis and even hepatocellular carcinoma (Dandri and Locarnini, 2012). Although the nucleos (t) ide analogs inhibited HBV-DNA and also reduced liver fibrosis for CHB patients, not all patients experienced improvement in liver fibrosis (Marcellin et al., 2013; Xu et al., 2015). The reason for this phenomenon may be associated with the following three aspects. First, activated hepatic stellate cell (HSC) secreted a variety of cytokines to maintain the continuity of activation. Second, disrupted liver micro-environment can lead to complex pathological effects between cell and cell and between cell and matrix. Third, the deposition of ECM degrades less. Therefore, except antiviral therapy, antifibrotic treatment is also necessary for CHB hepatic fibrosis (Sun et al., 2018).

Many TCM products against liver fibrosis have been widely applied in practice. On one hand, FZHY prescription, a representative one of FZHY therapy, can effectively improve liver fibrosis. Its mechanism included inhibiting the activation of HSC(Jiang et al., 2003), protecting liver cells from peroxidation and apoptosis (Hu et al., 1997), regulating hepatic ECM metabolism and angiogenesis (Liu et al., 2019), and adjusting differential expression at the molecular biological level of liver fibrosis (Xie et al., 2013). On the other hand, DHZC, a representative one of PYXZ, can reduce serum levels of transforming growth factor β1 and tumor necrosis factor-α by downregulating protein levels of phosphatidylinositol 3-kinase (PI3K) and phosphorylated Akt in the rat model with liver fibrosis, and *in vitro* experiment further confirmed that it was capable of suppressing HSC proliferation *via* downregulating PI3K/Akt (Gong et al., 2020). In addition to the earlier, a meta-analysis showed DHZC also improved CHB-related liver fibrosis by reducing serum biomarkers just like HA, LN, PC-III, and IV-C (Wei et al., 2015). Other than that, the combined use of ALHX (a representative one of RJSJ) with ETV could improve liver histology of CHB and boost the improvement rate of liver fibrosis (Jiang et al., 2012; Miao et al., 2019). All the aforementioned herbal formulas have been put into clinical application for many years and have obtained satisfactory treatment results for the patients.

There were several limitations, which need to be noticed in this NMA. First, all the RCTs included were conducted in China, which caused geographically limited distribution and thus the universality of therapeutic schedules should be cautious. Second, although we tried our best to set a classification criteria for different treatment strategies in case of heterogeneity, different documents with different sample sizes had various methods of administration (such as q.d., b.i.d., t.i.d.), which could be one of the sources of heterogeneity. Meanwhile, disproportion on quantity among treatment strategies may fluctuate the strength of evidence. Third, in the absence of the implementation of blinding or allocation concealment, this inadequate information could be the source of moderate methodological quality and “Low” quality of estimates. Finally, although the gender ratio of included patients remained balanced, it is noteworthy that in some RCTs, age differences among them may have contributed to the heterogeneity.

In conclusion, evidence from this NMA showed FZHY or combined with ETV had preferable effects in improving the clinical efficacy, recovering the level of serum biomarkers of live fibrosis (HA, IV-C) and serum parameters for liver function (ALT, ALB, and TBil), relieving the TCM symptoms (hypochondriac pain and poor appetite), regaining the width of portal vein and thickness of the spleen, and lessening side effects. Apart from these, ZYSG or combined with ETV could also be suitable to regain the level of serum biomarkers of live fibrosis (LN and PC-III) and serum parameters for liver function (AST), relieve the TCM symptom (fatigue) and HBV-DNA conversion. However, more relevant high-quality clinical articles should be acquired to strengthen the strength of existing evidence in the future.

## Data Availability

The original contributions presented in the study are included in the article/Supplementary Material; further inquiries can be directed to the corresponding authors.

## Abbreviations

TCMs, traditional Chinese medicines; CHB, chronic hepatitis B; NMA, network meta-analysis; SUCRA, surface under the cumulative ranking curve; HBV, hepatitis B virus; ECM, extracellular matrix; ETV, entecavir; FZHY prescription, Fuzheng Huayu prescription; DHZC, Dahuang Zhechong pill; ALHX, Anluo Huaxian pill; PRISRMA, the Preferred Reporting Items for Systematic Review and Meta-Analysis; CNKI, China National Knowledge Infrastructure; RCTs, randomized controlled trials; GRADE, the Grading of Recommendations Assessment, Development, and Evaluation; MCMC, the Markov chain Monte Carlo; SMD, standardized mean difference; RR, relative risk; CIs, confidence intervals; FZHY, Fuzheng Huayu therapy; HXHY, Huoxue Huayu therapy; RJSJ, Ruanjian Sanjie therapy; ZYSG, Ziyin Shugan therapy; SGHY, Shugan Huayu therapy; PYXZ, Poyu Xiaozheng therapy; HA, hyaluronic acid; LN, laminin; PC-III, pro-collagen type III; IV-C, collagen-IV; ALT, alanine aminotransferase; AST, aspartate aminotransferase; ALB, albumin; TBil, total bilirubin; HBV-DNA, hepatitis B virus-deoxyribonucleic acid; APRI, aminotransferase-to-platelet ratio index; HSC, hepatic stellate cell; and PI3K, phosphatidylinositol 3-kinase.
