# Supplementary material for: Comparison on different traditional Chinese medicine therapies for chronic hepatitis B liver fibrosis
Source: Front Pharmacol. 2022 Aug 10;13:943063. doi: 10.3389/fphar.2022.943063 (PMC9399442; doi:10.3389/fphar.2022.943063)
Supplement: Supplementary file 1 [file DataSheet1.ZIP › Supporting Informations/S4. Node-splitting analysis for outcomes.docx]

**Node-splitting analysis for outcomes**

**1. Clinical efficacy**

| Side | Direct | | Indirect | | Difference | | | tau |
| --- | --- | --- | --- | --- | --- | --- | --- | --- |
|  | Coef. | Std.Err. | Coef. | Std.Err. | Coef. | Std.Err. | P>\|z\| |  |
| D H* | -1.711071 | 0.7250885 | -0.4662926 | 186.4022 | -1.244778 | 186.4036 | 0.995 | 0.4193052 |
| A I * | -1.070147 | 0.2869549 | -3.889179 | 598.1599 | 2.819032 | 598.1607 | 0.996 | 0.4193048 |
| B I * | -1.679669 | 0.4671377 | -4.041876 | 864.3536 | 2.362207 | 864.3536 | 0.998 | 0.4193022 |
| C I * | -1.283588 | 0.4152882 | -3.902408 | 818.6117 | 2.61882 | 818.6117 | 0.997 | 0.4193041 |
| E F * | -1.485869 | 0.8043478 | -1.355522 | 1826.283 | -0.130347 | 1826.283 | 1.000 | 0.4193033 |
| F H * | -1.232144 | 0.6088172 | -2.509091 | 190.9392 | 1.276947 | 190.9402 | 0.995 | 0.4193055 |
| F I * | -1.396378 | 0.7426345 | -0.0474133 | 201.5389 | -1.348965 | 201.5402 | 0.995 | 0.4193052 |
| G I * | -1.457368 | 0.7519844 | -4.05996 | 1541.422 | 2.602591 | 1541.422 | 0.999 | 0.4193043 |

**Annotations:** A:RJSJ+ETV; B:ZYSG+ETV; C:FZHY+ETV; D: FZHY; E: HXHY; F: RJSJ; G:SGHY+ETV H: PYXZ; I: ETV.

1. **Hyaluronic acid**

| Side | Direct | | Indirect | | Difference | | | tau |
| --- | --- | --- | --- | --- | --- | --- | --- | --- |
|  | Coef. | Std.Err. | Coef. | Std.Err. | Coef. | Std.Err. | P>\|z\| |  |
| A L* | 106.13 | 44.97372 | 20.57102 | 13625.27 | 85.55899 | 13625.35 | 0.995 | 44.5394 |
| B L* | 27.81485 | 16.99919 | 20.54203 | 3908.341 | 7.272818 | 3908.378 | 0.999 | 44.54051 |
| C L* | 89.14298 | 22.62509 | 20.7805 | 10135.97 | 68.36248 | 10136 | 0.995 | 44.53947 |
| D L* | 29.31601 | 20.08695 | 20.45193 | 3714.767 | 8.864086 | 3714.822 | 0.998 | 44.54058 |
| F H* | 108.9 | 46.22545 | -66.19638 | 38345.95 | 175.0964 | 38345.98 | 0.996 | 44.53924 |
| G H* | 47 | 47.16547 | -66.3771 | 36266.3 | 113.3771 | 36266.34 | 0.998 | 44.53929 |
| H K | 185.77 | 40.75528 | 47.9409 | 51.90778 | 137.8291 | 65.99554 | 0.037 | 39.94221 |
| H L* | 43.45002 | 46.17999 | 54.11469 | 1145.047 | -10.66467 | 1145.978 | 0.993 | 44.54243 |
| I L* | 78.32 | 45.13172 | 20.55586 | 17929.63 | 57.76414 | 17929.69 | 0.997 | 44.53936 |
| J L* | 68.0339 | 31.55923 | 20.61664 | 5394.209 | 47.41725 | 5394.301 | 0.993 | 44.54002 |

**Annotations:** A:HXHY+ETV; B:RJSJ+ETV; C:ZYSG+ETV; D:FZHY+ETV; F:HXHY; G:HXHY+RJSJ; H:RJSJ; I:ZYSG; J:SGHY+ETV; K: PYXZ; L: ETV.

1. **Laminin**

| Side | Direct | | Indirect | | Difference | | | tau |
| --- | --- | --- | --- | --- | --- | --- | --- | --- |
|  | Coef. | Std.Err. | Coef. | Std.Err. | Coef. | Std.Err. | P>\|z\| |  |
| A L* | 8.760002 | 28.33329 | 103.0902 | 8635.431 | -94.33018 | 8635.478 | 0.991 | 28.06498 |
| B L* | 25.56624 | 10.80654 | 103.0963 | 3344.852 | -77.53005 | 3344.87 | 0.982 | 28.06528 |
| C L* | 59.7991 | 14.53201 | 103.3148 | 9490.384 | -43.51565 | 9490.395 | 0.996 | 28.06503 |
| D L* | 22.25766 | 12.66596 | 102.8419 | 3559.598 | -80.58426 | 3559.621 | 0.982 | 28.06529 |
| F H* | 8.699999 | 29.84517 | -21.11045 | 31983.72 | 29.81044 | 31983.74 | 0.999 | 28.06497 |
| G H* | 19 | 29.4473 | -21.24061 | 21823.92 | 40.24061 | 21823.94 | 0.999 | 28.06499 |
| H K | 117.92 | 25.74314 | 11.11682 | 28.06105 | 106.8032 | 38.08059 | 0.005 | 23.56738 |
| H L* | 62.19 | 30.14018 | 24.5074 | 1056.889 | 37.6826 | 1057.319 | 0.972 | 28.06587 |
| I L* | 46.9 | 28.35173 | 103.0725 | 10068.1 | -56.17253 | 10068.15 | 0.996 | 28.06504 |
| J L* | 43.53258 | 19.99574 | 103.1795 | 6897.671 | -59.6469 | 6897.701 | 0.993 | 28.06507 |

**Annotations:** A:HXHY+ETV; B:RJSJ+ETV; C:ZYSG+ETV; D:FZHY+ETV; F:HXHY; G:HXHY+RJSJ; H:RJSJ; I:ZYSG; J:SGHY+ETV; K: PYXZ; L: ETV.

1. **Pro-collagen type III**

| Side | Direct | | Indirect | | Difference | | | tau |
| --- | --- | --- | --- | --- | --- | --- | --- | --- |
|  | Coef. | Std.Err. | Coef. | Std.Err. | Coef. | Std.Err. | P>\|z\| |  |
| A L* | 2.97 | 32.58917 | 48.22506 | 1082.58 | -45.25506 | 1083.07 | 0.967 | 32.58534 |
| B L* | 27.30797 | 12.54346 | 48.02728 | 1743.924 | -20.71931 | 1743.969 | 0.991 | 32.57878 |
| C L* | 56.70018 | 16.82266 | 48.33214 | 9783.105 | 8.368042 | 9783.12 | 0.999 | 32.57645 |
| D L* | 32.88058 | 14.7602 | 47.84932 | 3405.629 | -14.96874 | 3405.662 | 0.996 | 32.57704 |
| F H* | -5.5 | 32.59995 | -5.106011 | 3858.534 | -0.3939895 | 3858.671 | 1.000 | 32.57682 |
| G H* | 22 | 33.37794 | -5.074696 | 17714.29 | 27.0747 | 17714.32 | 0.999 | 32.57638 |
| H K | 112.75 | 32.37379 | -10.6591 | 32.34676 | 123.4091 | 45.76434 | 0.007 | 27.83805 |
| H L* | 26.66 | 34.384 | 8.964539 | 434.9254 | 17.69546 | 436.2825 | 0.968 | 32.58682 |
| I L* | 84.78 | 33.02172 | 47.99516 | 13078.29 | 36.78484 | 13078.33 | 0.998 | 32.57643 |
| J L* | 45.2822 | 23.12013 | 48.05193 | 5361.511 | -2.769732 | 5361.561 | 1.000 | 32.57666 |

**Annotations:** A:HXHY+ETV; B:RJSJ+ETV; C:ZYSG+ETV; D:FZHY+ETV; F:HXHY; G:HXHY+RJSJ; H:RJSJ; I:ZYSG; J:SGHY+ETV; K: PYXZ; L: ETV.

1. **Collagen-IV**

| Side | Direct | | Indirect | | Difference | | | tau |
| --- | --- | --- | --- | --- | --- | --- | --- | --- |
|  | Coef. | Std.Err. | Coef. | Std.Err. | Coef. | Std.Err. | P>\|z\| |  |
| E H* | 33.02679 | 16.80727 | -6.154006 | 991.7199 | 39.18079 | 991.8589 | 0.968 | 17.74752 |
| A L* | 25.18 | 18.13416 | 107.5335 | 8203.59 | -82.3535 | 8203.611 | 0.992 | 17.74748 |
| B L* | 22.27326 | 6.971647 | 107.5244 | 3768.742 | -85.2511 | 3768.748 | 0.982 | 17.74749 |
| C L* | 42.61383 | 9.612378 | 107.0079 | 9051.397 | -64.39403 | 9051.402 | 0.994 | 17.74746 |
| D L* | 20.25077 | 8.194612 | 107.3906 | 3150.653 | -87.13981 | 3150.664 | 0.978 | 17.74752 |
| F H* | 21 | 18.40799 | 66.03584 | 15415.77 | -45.03584 | 15415.78 | 0.998 | 17.74746 |
| G H* | 16 | 18.61735 | 66.04748 | 13562.82 | -50.04748 | 13562.83 | 0.997 | 17.74746 |
| H K | 21.39999 | 18.78187 | -57.1625 | 17603.06 | 78.56249 | 17603.06 | 0.996 | 17.74746 |
| H L* | 20.76 | 18.94548 | -19.10158 | 1022.117 | 39.86158 | 1022.292 | 0.969 | 17.74754 |
| I L* | 36.1 | 17.95317 | 107.5345 | 7040.503 | -71.4345 | 7040.526 | 0.992 | 17.74759 |
| J L* | 71.21999 | 17.93267 | 107.5273 | 7005.123 | -36.30731 | 7005.147 | 0.996 | 17.7475 |

**Annotations:** A:HXHY+ETV; B:RJSJ+ETV; C:ZYSG+ETV; D:FZHY+ETV; E:FZHY; F:HXHY; G:HXHY+RJSJ; H:RJSJ; I:ZYSG; J:SGHY+ETV; K: PYXZ; L: ETV.

1. **Alanine aminotransferase**

| Side | Direct | | Indirect | | Difference | | | tau |
| --- | --- | --- | --- | --- | --- | --- | --- | --- |
|  | Coef. | Std.Err. | Coef. | Std.Err. | Coef. | Std.Err. | P>\|z\| |  |
| E I* | 19.38 | 8.592378 | -2.347132 | 542.0094 | 21.72713 | 542.0775 | 0.968 | 7.920906 |
| A J* | 9.389999 | 8.357711 | 56.6428 | 5918.468 | -47.2528 | 5918.474 | 0.994 | 7.920638 |
| B J* | 12.70377 | 3.31464 | 56.68525 | 1811.506 | -43.98148 | 1811.509 | 0.981 | 7.92074 |
| C J* | 12.76076 | 4.75223 | 56.44913 | 2987.649 | -43.68837 | 2987.653 | 0.988 | 7.920682 |
| D J* | 10.20362 | 3.667976 | 56.61051 | 1830.706 | -46.40689 | 1830.71 | 0.980 | 7.920751 |
| F I* | 4.65 | 8.314439 | 26.49584 | 545.7263 | -21.84584 | 545.7897 | 0.968 | 7.920895 |
| F J* | 13.62 | 9.022849 | -8.225775 | 547.5758 | 21.84578 | 547.6501 | 0.968 | 7.920905 |
| G J* | 20.11 | 8.409002 | 56.6731 | 7376.548 | -36.5631 | 7376.553 | 0.996 | 7.920652 |
| H J* | 26.54732 | 5.80784 | 56.81504 | 3761.211 | -30.26772 | 3761.216 | 0.994 | 7.920669 |

**Annotations:** A:HXHY+ETV; B:RJSJ+ETV; C:ZYSG+ETV; D:FZHY+ETV; E:FZHY; F:HXHY; G:HXHY+RJSJ; H:RJSJ; I:ZYSG; J:SGHY+ETV; K: PYXZ; L: ETV.

1. **Aspartate aminotransferase**

| Side | Direct | | Indirect | | Difference | | | tau |
| --- | --- | --- | --- | --- | --- | --- | --- | --- |
|  | Coef. | Std.Err. | Coef. | Std.Err. | Coef. | Std.Err. | P>\|z\| |  |
| C G* | 6.850229 | 4.369822 | 6.579732 | 532.6252 | 0.2704973 | 532.6431 | 1.000 | 9.544926 |
| A G* | 11.59345 | 4.374848 | 13.69297 | 1310.919 | -2.099515 | 1310.927 | 0.999 | 9.544916 |
| B G* | 15.691 | 5.591743 | 13.54898 | 2314.33 | 2.142023 | 2314.337 | 0.999 | 9.544885 |
| D G* | 17.29 | 12.63077 | 13.52292 | 25059.07 | 3.767077 | 25059.08 | 1.000 | 9.544859 |
| E G* | 8.93 | 9.746912 | 13.70381 | 5144.302 | -4.773811 | 5144.312 | 0.999 | 9.544875 |
| F G* | 20.97544 | 6.898367 | 13.75356 | 3717.668 | 7.221876 | 3717.675 | 0.998 | 9.544878 |

**Annotations:** A:RJSJ+ETV; B:ZYSG+ETV; C:FZHY+ETV; D:RJSJ; E:ZYSG; F:SGHY+ETV; G:ETV.

1. **Albumin**

| Side | Direct | | Indirect | | Difference | | | tau |
| --- | --- | --- | --- | --- | --- | --- | --- | --- |
|  | Coef. | Std.Err. | Coef. | Std.Err. | Coef. | Std.Err. | P>\|z\| |  |
| C G* | -1.038193 | 1.09683 | -2.654695 | 582.1549 | 1.616502 | 582.156 | 0.998 | 2.097534 |
| A G* | -4.990002 | 2.681914 | -2.072008 | 4657.441 | -2.917994 | 4657.442 | 1.000 | 2.097531 |
| B G* | -4.747272 | 1.823929 | -2.049128 | 2273.195 | -2.698144 | 2273.196 | 0.999 | 2.097531 |
| D G* | -4.049999 | 2.32986 | -2.072627 | 3184.827 | -1.977373 | 3184.828 | 1.000 | 2.097532 |
| E G* | -6.239998 | 2.244868 | -2.074785 | 2111.733 | -4.165212 | 2111.734 | 0.998 | 2.097533 |
| F G* | -5.5 | 2.263543 | -2.074415 | 2431.797 | -3.425585 | 2431.798 | 0.999 | 2.097533 |

**Annotations:** A:RJSJ+ETV; B:ZYSG+ETV; C:FZHY+ETV; D:RJSJ; E:ZYSG; F:SGHY+ETV; G:ETV.

1. **Total bilirubin**

| Side | Direct | | Indirect | | Difference | | | tau |
| --- | --- | --- | --- | --- | --- | --- | --- | --- |
|  | Coef. | Std.Err. | Coef. | Std.Err. | Coef. | Std.Err. | P>\|z\| |  |
| E I* | 19.5 | 3.82845 | -0.8748458 | 335.7342 | 20.37485 | 335.756 | 0.952 | 3.496087 |
| A J* | 4.9 | 3.760346 | 47.36486 | 3049.836 | -42.46486 | 3049.838 | 0.989 | 3.495994 |
| B J* | 9.541396 | 1.974599 | 47.39023 | 1327.803 | -37.84883 | 1327.805 | 0.977 | 3.49603 |
| C J* | 8.222398 | 2.562439 | 47.45938 | 1852.329 | -39.23698 | 1852.331 | 0.983 | 3.496001 |
| D J* | 1.79878 | 1.818401 | 47.36811 | 1150.944 | -45.56933 | 1150.945 | 0.968 | 3.496027 |
| F I* | 5.310002 | 3.847982 | 25.79794 | 337.2086 | -20.48794 | 337.2305 | 0.952 | 3.496089 |
| F J* | 9.5 | 4.255285 | -10.99564 | 339.1011 | 20.49564 | 339.1277 | 0.952 | 3.496103 |
| G J* | 25.3 | 4.365165 | 47.35251 | 6812.794 | -22.0525 | 6812.795 | 0.997 | 3.495988 |
| H J* | 8.899999 | 3.537326 | 47.36883 | 1519.605 | -38.46884 | 1519.609 | 0.980 | 3.496016 |

**Annotations:** A:HXHY+ETV; B:RJSJ+ETV; C:ZYSG+ETV; D:FZHY+ETV; E:FZHY; F:HXHY; G:HXHY+RJSJ; H:RJSJ; I:ZYSG; J:SGHY+ETV; K: PYXZ; L: ETV.

1. **Total TCM symptom scores**

| Side | Direct | | Indirect | | Difference | | | tau |
| --- | --- | --- | --- | --- | --- | --- | --- | --- |
|  | Coef. | Std.Err. | Coef. | Std.Err. | Coef. | Std.Err. | P>\|z\| |  |
| D E* | 0.581464 | 0.605935 | -3.934483 | 122.4048 | 4.515947 | 122.4063 | 0.971 | 0.4918942 |
| A G* | 1.478284 | 0.3149811 | 12.02132 | 418.8969 | -10.54303 | 418.8971 | 0.980 | 0.4918863 |
| B G* | 14 | 1.705469 | 11.92428 | 3757.122 | 2.075725 | 3757.122 | 1.000 | 0.4918929 |
| C G* | 3.162244 | 0.3178554 | 11.97003 | 314.8239 | -8.807784 | 314.8242 | 0.978 | 0.4918939 |
| E G* | 5.429999 | 0.9311548 | 0.8971317 | 122.8437 | 4.532868 | 122.8473 | 0.971 | 0.4918936 |
| F G* | 10.97 | 0.662529 | 12.02138 | 1242.59 | -1.051376 | 1242.591 | 0.999 | 0.491891 |

**Annotations:** A:RJSJ+ETV; B:ZYSG+ETV; C:FZHY+ETV; D:FZHY; E:RJSJ; F:SGHY+ETV; E:ETV.

1. **Hypochondriac pain**

| Side | Direct | | Indirect | | Difference | | | tau |
| --- | --- | --- | --- | --- | --- | --- | --- | --- |
|  | Coef. | Std.Err. | Coef. | Std.Err. | Coef. | Std.Err. | P>\|z\| |  |
| C E* | 0.7525359 | 0.2098604 | 0.2848467 | 40.25824 | 0.4676892 | 40.25879 | 0.991 | 0.2911652 |
| A E* | 0.4095491 | 0.2131076 | 1.505578 | 101.8512 | -1.096029 | 101.8514 | 0.991 | 0.291165 |
| B E* | 0.8456353 | 0.1777913 | 1.511011 | 134.8001 | -0.6653758 | 134.8002 | 0.996 | 0.2911634 |
| D E* | 0.8099999 | 0.3936517 | 1.503235 | 610.2433 | -0.6932352 | 610.2435 | 0.999 | 0.2911631 |

**Annotations:** A:RJSJ+ETV; B:ZYSG+ETV; C:FZHY+ETV; D:ZYSG; E:ETV.

1. **Poor appetite**

| Side | Direct | | Indirect | | Difference | | | tau |
| --- | --- | --- | --- | --- | --- | --- | --- | --- |
|  | Coef. | Std.Err. | Coef. | Std.Err. | Coef. | Std.Err. | P>\|z\| |  |
| C E* | 0.6978839 | 0.155163 | 0.3919396 | 118.4398 | 0.3059443 | 118.4399 | 0.998 | 0.2096463 |
| A E* | 0.64 | 0.2452686 | 1.395099 | 381.1272 | -0.7550986 | 381.1273 | 0.998 | 0.2096485 |
| B E* | 0.7 | 0.2453206 | 1.405101 | 349.2742 | -0.7051012 | 349.2743 | 0.998 | 0.2096467 |
| D E* | 1.52 | 0.3254582 | 1.395771 | 649.8938 | 0.1242285 | 649.8939 | 1.000 | 0.2096464 |

**Annotations:** A:RJSJ+ETV; B:ZYSG+ETV; C:FZHY+ETV; D:ZYSG; E:ETV.

1. **Fatigue**

| Side | Direct | | Indirect | | Difference | | | tau |
| --- | --- | --- | --- | --- | --- | --- | --- | --- |
|  | Coef. | Std.Err. | Coef. | Std.Err. | Coef. | Std.Err. | P>\|z\| |  |
| C F* | 0.6733779 | 0.2472545 | 0.3372685 | 46.08773 | 0.3361094 | 46.08839 | 0.994 | 0.3459897 |
| A F* | 0.81 | 0.3794441 | 1.346888 | 478.5182 | -0.536888 | 478.5184 | 0.999 | 0.3459869 |
| B F* | 0.5560907 | 0.2077433 | 1.346423 | 129.6045 | -0.7903322 | 129.6047 | 0.995 | 0.3459884 |
| D F* | 0.91 | 0.4495916 | 1.346736 | 685.3789 | -0.4367357 | 685.3791 | 0.999 | 0.3459868 |
| E F* | 0.79 | 0.3494961 | 1.34685 | 139.1198 | -0.5568504 | 139.1202 | 0.997 | 0.3459892 |

**Annotations:** A:RJSJ+ETV; B:ZYSG+ETV; C:FZHY+ETV; D:ZYSG; E:SGHY+ETV; F:ETV.

1. **Thickness of spleen**

| Side | Direct | | Indirect | | Difference | | | tau |
| --- | --- | --- | --- | --- | --- | --- | --- | --- |
|  | Coef. | Std.Err. | Coef. | Std.Err. | Coef. | Std.Err. | P>\|z\| |  |
| C D* | 0.5999997 | 0.6726188 | -0.347738 | 105.9211 | 0.9477377 | 105.9232 | 0.993 | 0.6684991 |
| A G* | 0.1000001 | 0.6851733 | 2.522884 | 411.6607 | -2.422884 | 411.6613 | 0.995 | 0.6684594 |
| B G* | 2.093168 | 0.6017829 | 2.425757 | 874.5889 | -0.3325888 | 874.589 | 1.000 | 0.6684411 |
| D G* | 0.6600007 | 0.6738008 | -0.6172989 | 149.819 | 1.2773 | 149.8205 | 0.993 | 0.6684935 |
| E G* | 3.84 | 1.097617 | 2.519778 | 2405.726 | 1.320222 | 2405.726 | 1.000 | 0.668436 |

**Annotations:** A:ZYSG+ETV; B:FZHY+ETV; C:FZHY; D:RJSJ; E:SGHY+ETV; G:ETV.

1. **Width of portal vein**

| Side | Direct | | Indirect | | Difference | | | tau |
| --- | --- | --- | --- | --- | --- | --- | --- | --- |
|  | Coef. | Std.Err. | Coef. | Std.Err. | Coef. | Std.Err. | P>\|z\| |  |
| C D* | 0.1700017 | 0.1700299 | -0.9727608 | 91.82272 | 1.142762 | 91.82287 | 0.990 | 0.1690715 |
| A G* | 0.5999994 | 0.3051698 | 3.147685 | 683.5795 | -2.547686 | 683.5795 | 0.997 | 0.1690674 |
| B G* | 0.7625349 | 0.1534809 | 3.109062 | 202.4893 | -2.346527 | 202.4895 | 0.991 | 0.1690709 |
| D G* | 1.4 | 0.3202882 | 0.2356529 | 94.34487 | 1.164347 | 94.34541 | 0.990 | 0.1690696 |
| E G* | 1.58 | 0.31786 | 3.139991 | 788.1116 | -1.559991 | 788.1117 | 0.998 | 0.1690673 |

**Annotations:** A:ZYSG+ETV; B:FZHY+ETV; C:FZHY; D:RJSJ; E:SGHY+ETV; G:ETV.

1. **HBV-DNA conversion rate**

| Side | Direct | | Indirect | | Difference | | | tau |
| --- | --- | --- | --- | --- | --- | --- | --- | --- |
|  | Coef. | Std.Err. | Coef. | Std.Err. | Coef. | Std.Err. | P>\|z\| |  |
| C G* | -1.126175 | 0.3003687 | -0.7535783 | 336.1548 | -0.3725963 | 336.1549 | 0.999 | 3.07e-07 |
| A G* | -0.0740114 | 0.1800571 | -2.282665 | 1358.265 | 2.208654 | 1358.265 | 0.999 | 6.80e-08 |
| B G* | -5.068904 | 1.464378 | -4.58608 | 1323.057 | -0.4828241 | 1323.057 | 1.000 | 3.02e-07 |
| D E* | -1.650681 | 0.7168759 | -1.397531 | 1518.753 | -0.25315 | 1518.754 | 1.000 | 2.04e-07 |
| E G* | -0.2451224 | 0.7028238 | -0.3385856 | 708.3225 | 0.0934632 | 708.3229 | 1.000 | 7.01e-07 |
| F G* | -1.504077 | 0.591608 | -2.499057 | 1394.48 | 0.9949796 | 1394.48 | 0.999 | 3.02e-07 |

**Annotations:** A:RJSJ+ETV; B:ZYSG+ETV; C:FZHY+ETV; D:HXHY+RJSJ; E:RJSJ; F:ZYSG; G:ETV.

1. **Rates of adverse reactions**

| Side | Direct | | Indirect | | Difference | | | tau |
| --- | --- | --- | --- | --- | --- | --- | --- | --- |
|  | Coef. | Std.Err. | Coef. | Std.Err. | Coef. | Std.Err. | P>\|z\| |  |
| C D* | 0.0006899 | 0.5273839 | 0.0177426 | 645.1484 | -0.0170526 | 645.1486 | 1.000 | 3.66e-08 |
| A D* | 0.2719337 | 0.7400129 | 0.013052 | 1630.047 | 0.2588817 | 1630.047 | 1.000 | 2.27e-08 |
| B D* | -0.2548922 | 0.7165948 | 0.013233 | 1884.679 | -0.2681253 | 1884.679 | 1.000 | 6.71e-08 |

**Annotations:** A:HXHY+ETV; B:RJSJ+ETV; C:FZHY+ETV; D:ETV.
