# Supplementary material for: Comparison on different traditional Chinese medicine therapies for chronic hepatitis B liver fibrosis
Source: Front Pharmacol. 2022 Aug 10;13:943063. doi: 10.3389/fphar.2022.943063 (PMC9399442; doi:10.3389/fphar.2022.943063)
Supplement: Supplementary file 1 [file DataSheet1.ZIP › Supporting Informations/S2. Search Strategies for each database.doc]

**Search strategies for each database**

**PubMed:**

#1 chronic hepatits B [SH]

#2 hepatitis B virus [SH]

#3 liver fibrosis [SH]

#4 hepatic fibrosis [SH]

#5 chronic hepatits B liver fibrosis [SH]

#6 #1 or #2 or #3 or #4 or #5

#7 traditional Chinese medicine [SH]

#8 Chinese medicine [SH]

#9 herbal medicine [SH]

#10 herbs [SH]

#11 exp Fuzheng Huayu [SH]

#12 exp Dahuang Zhechong [SH]

#13 exp Anluo Huaxian [SH]

#14 exp Biejia Ruangan [SH]

#15 antiviral drugs [SH]

#16 antiviral agents [SH]

#17 entecavir [SH]

#18 adefovir [SH]

#19 #6 or #7 or #8 or #9 or #10 or #11 or #12 or #13 or #14 or #15 or #16 or #17 or #18

#20 randomized controlled trial [PT]

#21 controlled clinical trial [PT]

#22 randomized [TIAB]

#23 #19 or #20 or #21 or #22

#24 #6 and #19 and #23

Annotation: Mesh Subheadings [SH] Publication Type [PT] Title/Abstract [TIAB]

**MEDLINE:**

1. exp chronic hepatits B liver fibrosis.mp.

2. (chronic hepatits B liver fibrosis OR chronic hepatits B OR hepatitis B virus OR liver fibrosis OR hepatic fibrosis) .tw.

3. 1 OR 2
4. exp traditional Chinese medicine.mp.
5. (Chinese medicine, herbs, herbal medicine, Fuzheng Huayu, Dahuang Zhechong OR Anluo Huaxian OR Biejia Ruangan).tw.
6. 4 OR 5
7. randomized controlled trial.pt.
8. controlled clinical trial.pt.
9. randomized.ti,ab.
10. randomly.ti,ab.
11. trial.ti,ab.
12. groups.ti,ab.
13. OR/7-12
14. 3 AND 6 AND 13

**EMBASE:**

1 (chronic hepatits B liver fibrosis or chronic hepatits B or hepatitis B virus or liver fibrosis or hepatic fibrosis).af.

2 (traditional Chinese medicine).af.

3 exp Chinese medicine/ or exp herbs/ or exp herbal medicine/ or exp Fuzheng Huayu/ or exp Dahuang Zhechong/ or exp Anluo Huaxian/ or exp Biejia Ruangan/

4 2 or 3

5 (random* OR factorial* OR crossover*).af.

6 exp crossover-procedure/ or exp double-blind procedure/ or exp randomized controlled trial/ or single-blind procedure/

7 5 or 6

8 4 and 7

**Scopus and Web of Science and Google Scholar:**

#1 traditional Chinese medicine OR Chinese medicine OR herbs OR herbal medicine OR Fuzheng Huayu OR Dahuang Zhechong OR Anluo Huaxian OR Biejia Ruangan

#2 chronic hepatits B liver fibrosis OR chronic hepatits B OR hepatitis B virus OR liver fibrosis OR hepatic fibrosis

#3 randomized controlled trial OR controlled clinical trial OR randomized

#4 #1 AND #2 AND #3

**CNKI** (China National Knowledge Infrastructure) and **WanFang DATA**:

#1 traditional Chinese medicine or Chinese medicine or herbs or herbal medicine or Fuzheng Huayu or Dahuang Zhechong or Anluo Huaxian or Biejia Ruangan

#2 chronic hepatits B liver fibrosis or chronic hepatits B or hepatitis B virus or liver fibrosis or hepatic fibrosis

#3 randomized controlled trial OR controlled clinical trial OR randomized

#4 #1 AND #2 AND #3
