# Supplementary material for: Comparison on different traditional Chinese medicine therapies for chronic hepatitis B liver fibrosis
Source: Front Pharmacol. 2022 Aug 10;13:943063. doi: 10.3389/fphar.2022.943063 (PMC9399442; doi:10.3389/fphar.2022.943063)
Supplement: Supplementary file 1 [file DataSheet1.ZIP › Supporting Informations/S3. The composition of four representative formulas.doc]

**S2. The composition of four representative formulas.**

| **Formulas** | **Ingredients of each formula** | | **Usage and dosage** |
| --- | --- | --- | --- |
|  | **Latin name** | **Chinese name** |  |
| **Fuzheng Huayu tablet/capsule** | Salvia miltiorrhiza Bge | Dan Shen | Po. 1.5g, three times a day |
|  | Cordyeps sinensis (Berk.) Sacc | Dongchong Xiacao |  |
|  | Prunus persica (L.) Batsch | Tao Ren |  |
|  | Pini Pollen | Songhua Fen |  |
|  | Gynostemma pentaphllam (Thunb.)Makino | Jiao Gulan |  |
|  | Schisandra chinesis (Turcz.) Baill | Wu Weizi |  |
| **Dahuang Zhechong pill** | Rheum palmatum L | Da Huang | Po. 3g, twice a day |
|  | Eupolyphaga sinensis Walk. | Tu Biechong |  |
|  | Whitemania pigra Whitman | Shui Zhi |  |
|  | Tabanus | Meng Chong |  |
|  | Holotrichia diomphalia Bates | Qi Cao |  |
|  | Toxicodendron vernicifluum | Gan Qi |  |
|  | Prunus persica (L.) Batsch | Tao Ren |  |
|  | Semen Armeniacae Amarum. | Ku Xingren |  |
|  | Scutellaria baicalensis Georg | Huang Qin |  |
|  | Rehmannia glutinosa Libosch. | Di Huang |  |
|  | Cynanchum otophyllum | Bai Shao |  |
|  | Radix Glycyrrhizae preparata | Gan Cao |  |
| **Anluo Huaxian pill** | Rehmannia glutinosa Libosch. | Di Huang | Po. 6g, twice a day |
|  | Panax notoginseng (Burk.) F. H. Chen | San Qi |  |
|  | Whitemania pigra Whitman | Shui Zhi |  |
|  | Bombyx mori Linnaeus. | Jiang Can |  |
|  | Pheretima aspergillum (E. Perrier) | Di Long |  |
|  | Atractylodes macrocephala Koidz. | Bai Zhu |  |
|  | Curcuma wenyujin Y.H.Chen et C.Ling | Yu Jin |  |
|  | Bos taurus domesticus Gmelin | Niu Huang |  |
|  | Arca subcrenata Lischke | Wa Lengzi |  |
|  | Paeonia suffruticosa Andr. | Mu Danpi |  |
|  | Rheum palmatum L | Da Huang |  |
|  | Hordeum vulgare L. | Raw Mai Ya |  |
|  | Gallus gallus domesticus Brisson | Ji Neijin |  |
|  | Bubalus bubalis Linnaeus | Shui Niujiao |  |
| **Biejia Ruangan tablet** | Trionyx sinensis Wiegmann | Bie Jia | Po. 4 tables, three times a day |
|  | Curcuma phaeocaulis Val. | E Zhu |  |
|  | Paeonia lactiflora Pall. | Chi Shao |  |
|  | Aaugellica sinensis(Oliv) Diels. | Dang Gui |  |
|  | Panax notoginseng (Burk.) F. H. Chen | San Qi |  |
|  | Codonopsis pilosula (Franch.) Nannf. | Dang Shen |  |
|  | Astragalus membranaceus | Huang Qi |  |
|  | Placenta Hominis | Zi Heche |  |
|  | Cordyeps sinensis (Berk.) Sacc | Dongchong Xiacao |  |
|  | lsatis indigotica Fort. | Ban Langen |  |
|  | Forsythia suspensa (Thunb.) Vahl | Lian Qiao |  |
